# Supplementary material for: CXCL10-induced chemotaxis of ex vivo-expanded natural killer cells combined with NKTR-255 enhances anti-tumor efficacy in osteosarcoma
Source: Mol Ther Oncol. 2025 Sep 11;33(4):201051. doi: 10.1016/j.omton.2025.201051 (PMC12509742; doi:10.1016/j.omton.2025.201051)
Supplement: Document S1. Figures S1–S7, Tables S1 and S2, and supplemental materials and methods [file mmc1.pdf]

OMTON, Volume *33*

Supplemental information

CXCL10-induced chemotaxis of *ex vivo*-expanded natural killer cells combined with NKTR-255 enhances anti-tumor efficacy in osteosarcoma

Shiori Eguchi, Wen Luo, Hongwen Zhu, Hai M. Hoang, Changxin Xu, Gregory K. Behbehani, Kazi L. Tasneem, Janet Ayello, Mario Marcondes, Dean A. Lee, and Mitchell S. Cairo

SUPPLEMENTAL MATERIALS AND METHODS

Immunohistochemical staining

The collected tissues were embedded in Tissue-Tec OCT Compound and snap-frozen on dry ice. For each specimen, 7 μm serial sections were cut and stored at -80 °C. Sections were fixed in freshly diluted 4% paraformaldehyde (Electron Microscopy Sciences, Hatfield, PA, USA) and blocked with M.O.M. blocking reagent with 0.1% Triton (Sigma, St. Louis, MO, USA). The slides were then incubated with an anti-human CD45 antibody (1:100 dilution; Thermo Fisher Scientific, Cat# 14-9457-82; RRID: AB_11063696) and an Alexa Fluor 488-conjugated secondary antibody (1:1000 dilution; Thermo Fisher Scientific, Cat# A-11001; RRID: AB_2534069), and mounted in a Vectashield mounting medium containing DAPI (Vector Laboratories, Newark, CA, USA). Images were acquired using the EVOS M5000 imaging system (Thermo Fisher Scientific; RRID: SCR_023650).

Mass cytometry

Antibody cocktails were prepared at The Ohio State University, frozen in aliquots at -80 °C, and shipped to New York Medical College on dry ice, where they were stored at -80 °C until tumors were harvested from the experimental mice and dissociated into single-cell suspensions. Cells (1-2 $\times$ 10^6) were washed once with cell staining medium (CSM) (PBS, 0.5% bovine serum albumin) and blocked with 5 μL of Fc block (BD Biosciences, Cat# 564219; RRID: AB_2728082) diluted in 50 μL of CSM at room temperature for 10 minutes. 45 μL of the surface antibody cocktail was then added and cells were incubated at 4 °C for 1 h with periodical mixing. Cells were washed twice with CSM and fixed with 1.5% paraformaldehyde (Millipore Sigma, Burlington, MA, USA) at room temperature for 15 minutes. Cells were washed once with CSM, vortexed for 1.5 minutes, immediately resuspended with 1 mL of ice-cold methanol (Sigma-Aldrich), and incubated at -20 °C for 15 minutes. Cells were collected by centrifugation, vortexed, washed three times with CSM, and stained with 50 μL of the intracellular antibody cocktail diluted in 50 μL of CSM at 4 °C for 1 h with periodical mixing. Cells were washed once with CSM, fixed with proteomic stabilizer PROT1 (Smart Tube, Inc., Las Vegas, NV, USA) at room temperature for 10 minutes, frozen in aliquots at -80 °C, and shipped back to The Ohio State University on dry ice, where they were stored at -80 °C until they were analyzed on a mass cytometer. Samples were thawed, intercalated with Ir (191/193) intercalator (Standard Biotools, South San Francisco, CA, USA), washed into pure water with normalization beads (Standard Biotools), and then run on the Fluidigm CyTOF Helios Mass Cytometer (Fluidigm, South San Francisco, CA, USA; RRID: SCR_019916) at approximately 150-300 cell events per second. All events (Table S1) were collected from each sample. Mean ion counts for each cell events were normalized using an automated script[1] and the resulting normalized FCS files were then uploaded for all subsequent analysis on the Cytobank platform. Singlet cells were gated on plots of event length vs. Ir and offset vs. residual as previously described.[2]

Table S1. Number of events analyzed by single-cell RNA sequencing and mass cytometry

Tumor	Treatment	Analyzed events
Single-cell RNA sequencing		
WT[a]	PBS[b]	739
	NK[c] + NKTR-255	787
CXCL10	PBS	408
	NK + NKTR-255	571
Mass cytometry		
WT	PBS	80,098
		184,998
		113,219
	NK + NKTR-255	75,172
		20,411
		174,033
CXCL10	PBS	46,490
		24,899
		73,996
	NK + NKTR-255	22,313
		44,351
		36,845

[a]WT, wild-type; [b]PBS, phosphate buffer solution; [c]NK, natural killer

Table S2. List of antibodies used in mass cytometry alongside their clone information, vendor, metal label, and concentration used

Antibody	Clone	Vendor	Metal label	Concentration (μg/mL)
hCD45	HI30	BioLegend	89-Y	2
GAPDH	6c5	Invitrogen	112-Sn	2
mCD45.1	30-F11	BioLegend	115-In	1
cPARP	F21-A52	BD Biosciences	140-Ce	2
Ly6G	1A8	BioLegend	141-Pr	1
mCD11b	M1/70	BioLegend	143-Nd	1
hCD94	HP-3D9	BD Biosciences	144-Nd	2
hCD56	NCAM16.2	BD Biosciences	147-Sm	1
TIGIT	A15153G	BioLegend	150-Nd	3
HLA-DR	L243	BioLegend	153-Eu	2
hCD69	FN50	BioLegend	154-Sm	2
mCD69	H1.2F3	BioLegend	155-Gd	2
Ki-67	SolA15	Invitrogen	158-Gd	4
hNKp30	P30-15	BioLegend	159-Tb	1
hCD16	3G8	BioLegend	161-Dy	2
mCD44	IM7	BioLegend	164-Dy	1
pRb	J112-906	BD Biosciences	165-Ho	1.5
NKG2D	1D11	BioLegend	166-Er	4
mCD206	C068C2	BioLegend	167-Er	2
mF4/80	BM8	BioLegend	168-Er	2
NKG2A	S19004C	BioLegend	169-Tm	2
hCD107a	H4A3	BioLegend	170-Er	2
hPerforin	dG9	BioLegend	175-Lu	4

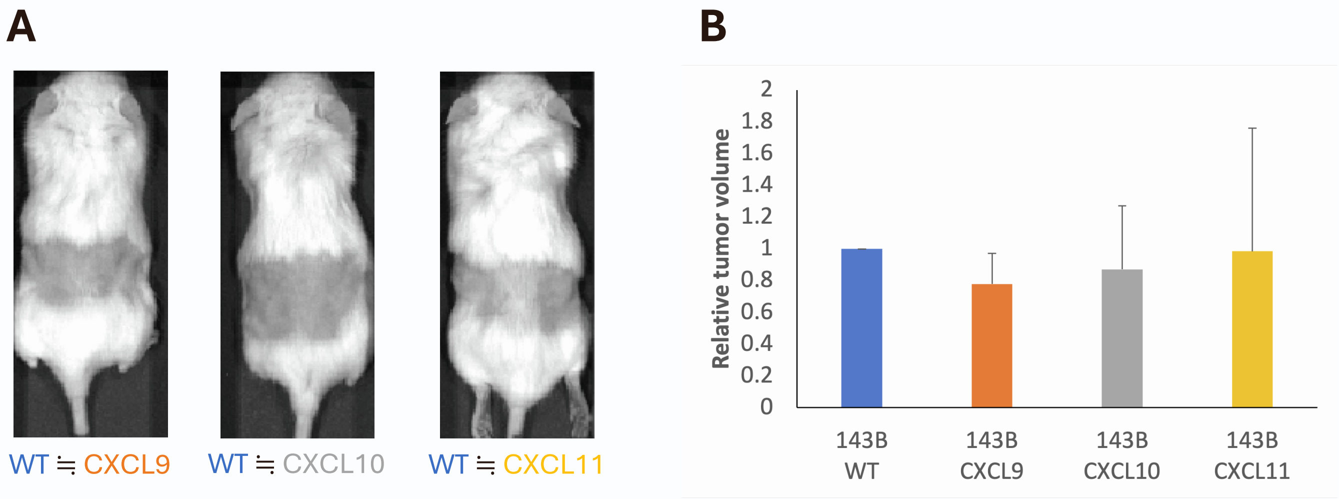

Figure S1. Similar sizes of WT and chemokine-secreting 143B tumors

NSG mice were inoculated s.c. with 2×10^6 WT and chemokine (either CXCL9, -10, or -11)-secreting 143B cells in each flank of the same mouse (n = 3 mice/group). Mice were imaged and tumor sizes were measured by a digital caliper 5 days following tumor inoculation.

(A) Example of tumor burden of a mouse inoculated with WT (left flank) and chemokine (either CXCL9, -10, or -11)-secreting 143B cells (right flank). One of the three representative mice/group is shown. (B) Volume of chemokine (either CXCL9, -10, or -11)-secreting tumors relative to WT tumors. Data are represented as mean ± SD. The comparison of WT and chemokine (CXCL9, -10, or -11)-secreting tumor volume was not statistically significant (Student's t test). WT, wild-type; NSG, NOD/SCID/gamma$^{-/-}$; s.c., subcutaneously; SD, standard deviation.

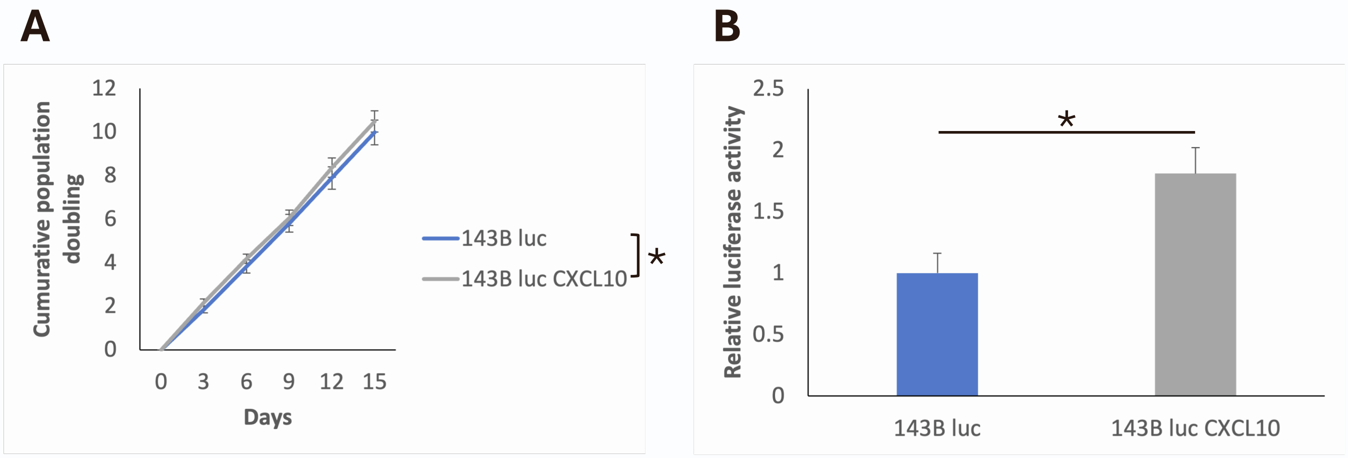

Figure S2. Significant differences in growth and luciferase activity between *CXCL10* virus-infected and non-infected 143B luc cells

(A) Growth curve of *CXCL10* virus-infected and non-infected 143B luc cells. Data are represented as mean ± SD of 3 independent biological replicates. *$p < 0.05$ (Student's t test). *CXCL10* virus-infected 143B luc cells grew significantly faster than non-infected 143B luc cells. (B) Luciferase activity of *CXCL10* virus-infected and non-infected 143B luc cells. Data are represented as mean ± SD of triplicates in a representative experiment. Experiments were repeated 3 times with similar results. *$p < 0.05$ (Student's t test). *CXCL10* virus-infected 143B luc cells had significantly higher luciferase activity than non-infected 143B luc cells. luc, luciferase-expressing; SD, standard deviation.

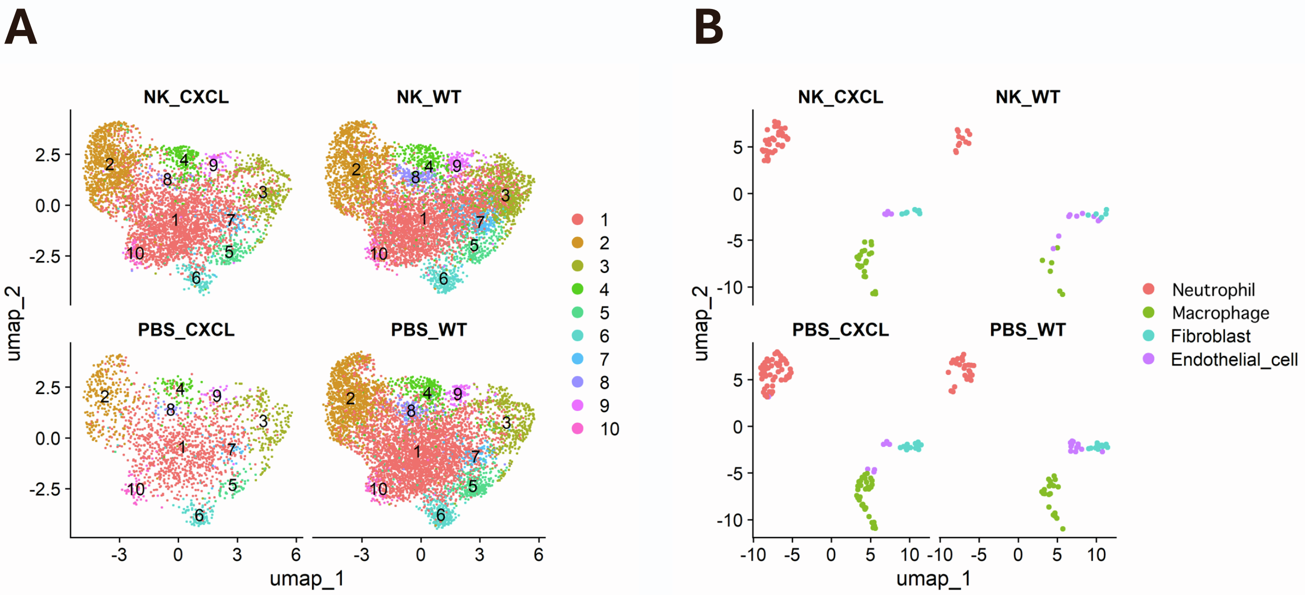

Figure S3. Clustering of all human and mouse cells

(A) UMAP displaying initial clustering of all human cells. (B) UMAP displaying initial clustering of all mouse cells. NK_CXCL, NK+NKTR-255-treated CXCL10-positive OSA; NK_WT, NK+NKTR-255-treated WT OSA; PBS_CXCL, PBS-treated CXCL10-positive OSA; PBS_WT, PBS-treated WT OSA; NK, natural killer; OSA, osteosarcoma; WT, wild-type; PBS, phosphate buffer solution; UMAP, uniform manifold approximation and projection.

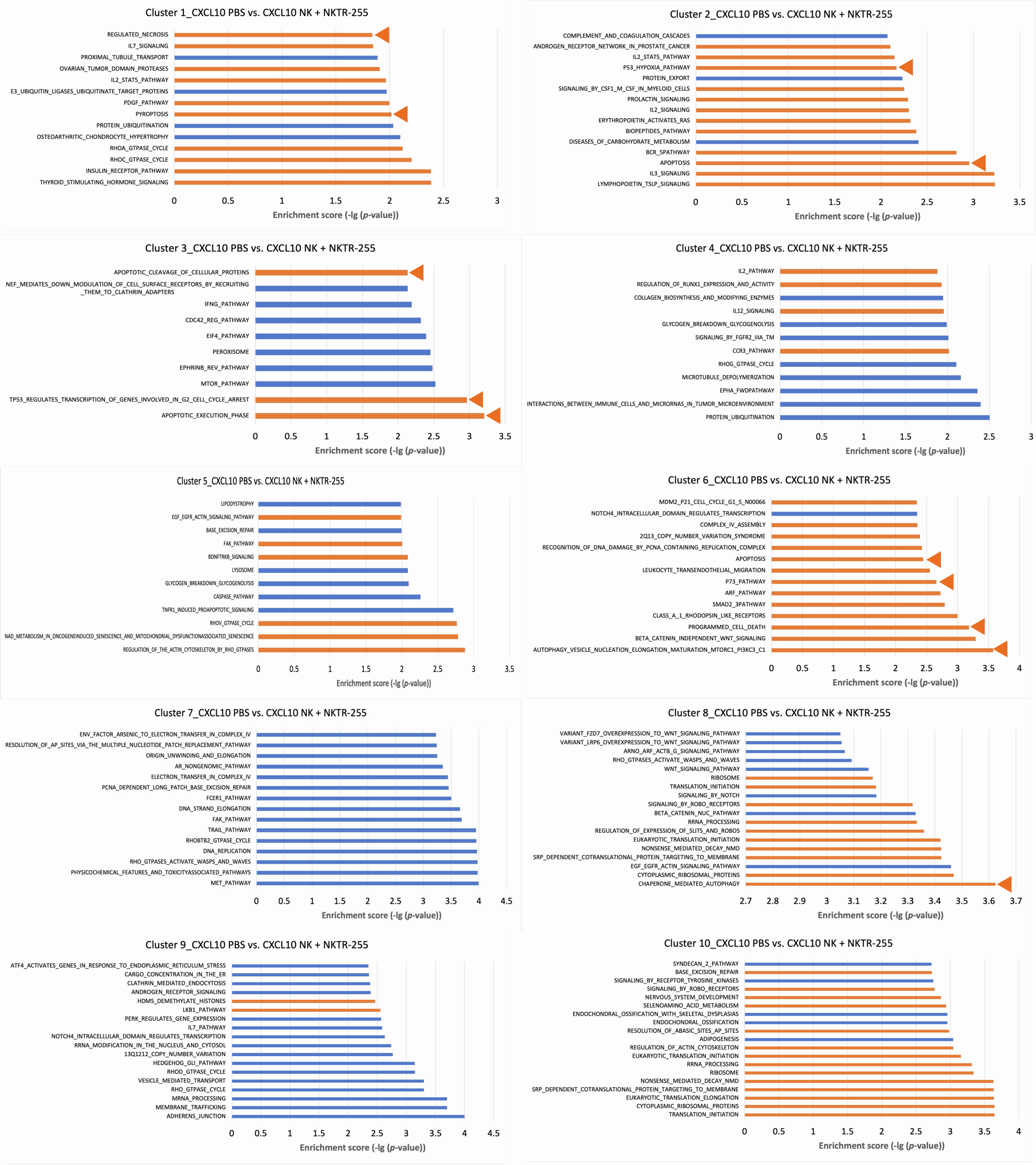

Figure S4. GSEA bar charts showing enrichment scores for the hallmark pathways that were different between CXCL10-positive tumors with and without treatment with NK cells and NKTR-255

Pathways upregulated in the CXCL10-positive tumor treated with NK cells and NKTR-255 were shown in orange. Pathways downregulated in the CXCL10-positive tumor treated with NK cells and NKTR-255 were shown in blue. Pathways indicated by arrowhead (◀) were related to cell death. GSEA, gene set enrichment analysis; NK, natural killer; PBS, phosphate buffer solution.

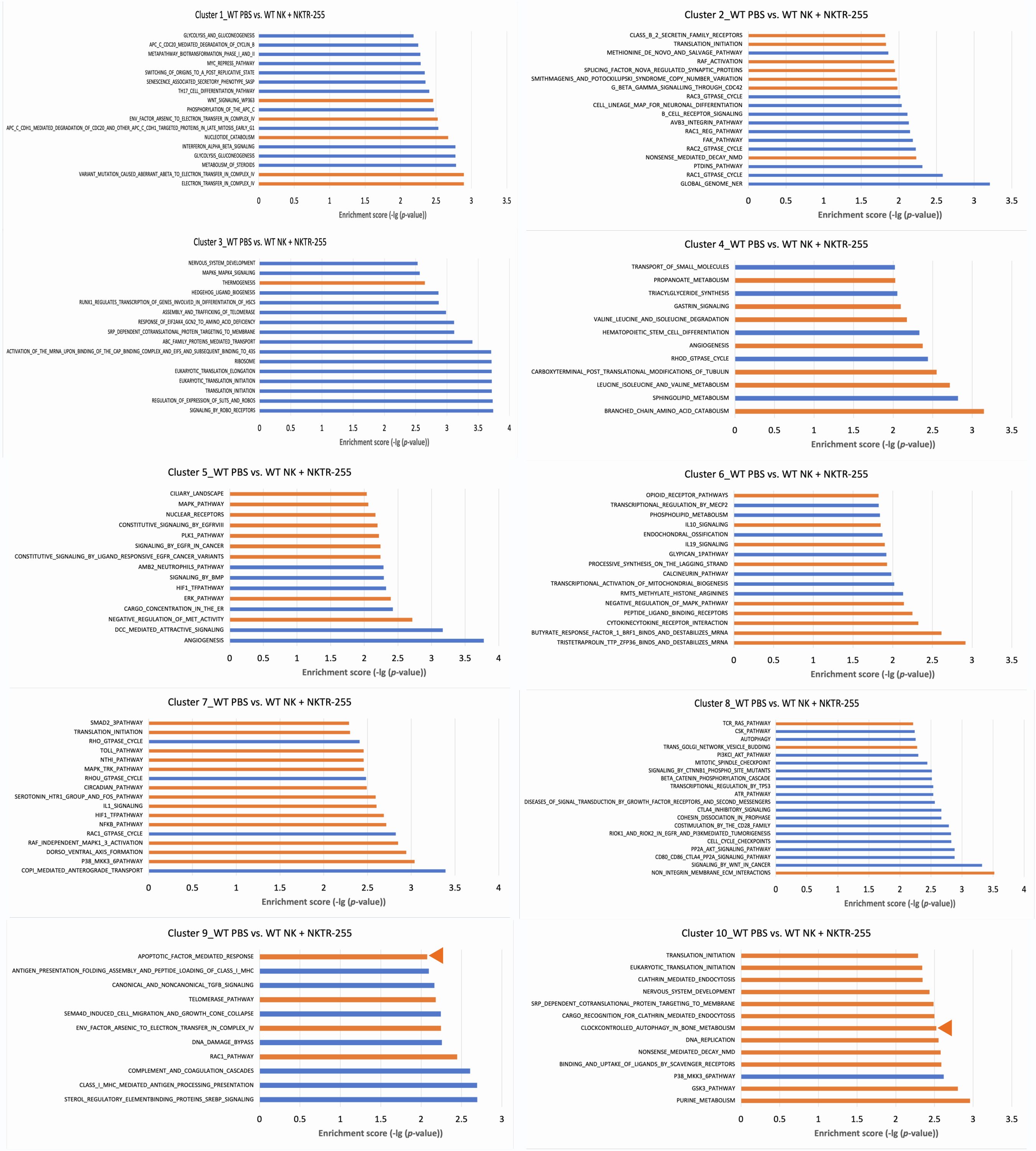

Figure S5. GSEA bar charts showing enrichment scores for the hallmark pathways that were different between WT tumors with and without treatment with NK cells and NKTR-255
Pathways upregulated in the WT tumor treated with NK cells and NKTR-255 were shown in orange. Pathways downregulated in the WT tumor treated with NK cells and NKTR-255 were shown in blue. Pathways indicated by arrowhead (◀) were related to cell death. GSEA, gene set enrichment analysis; WT, wild-type; NK, natural killer; PBS, phosphate buffer solution.

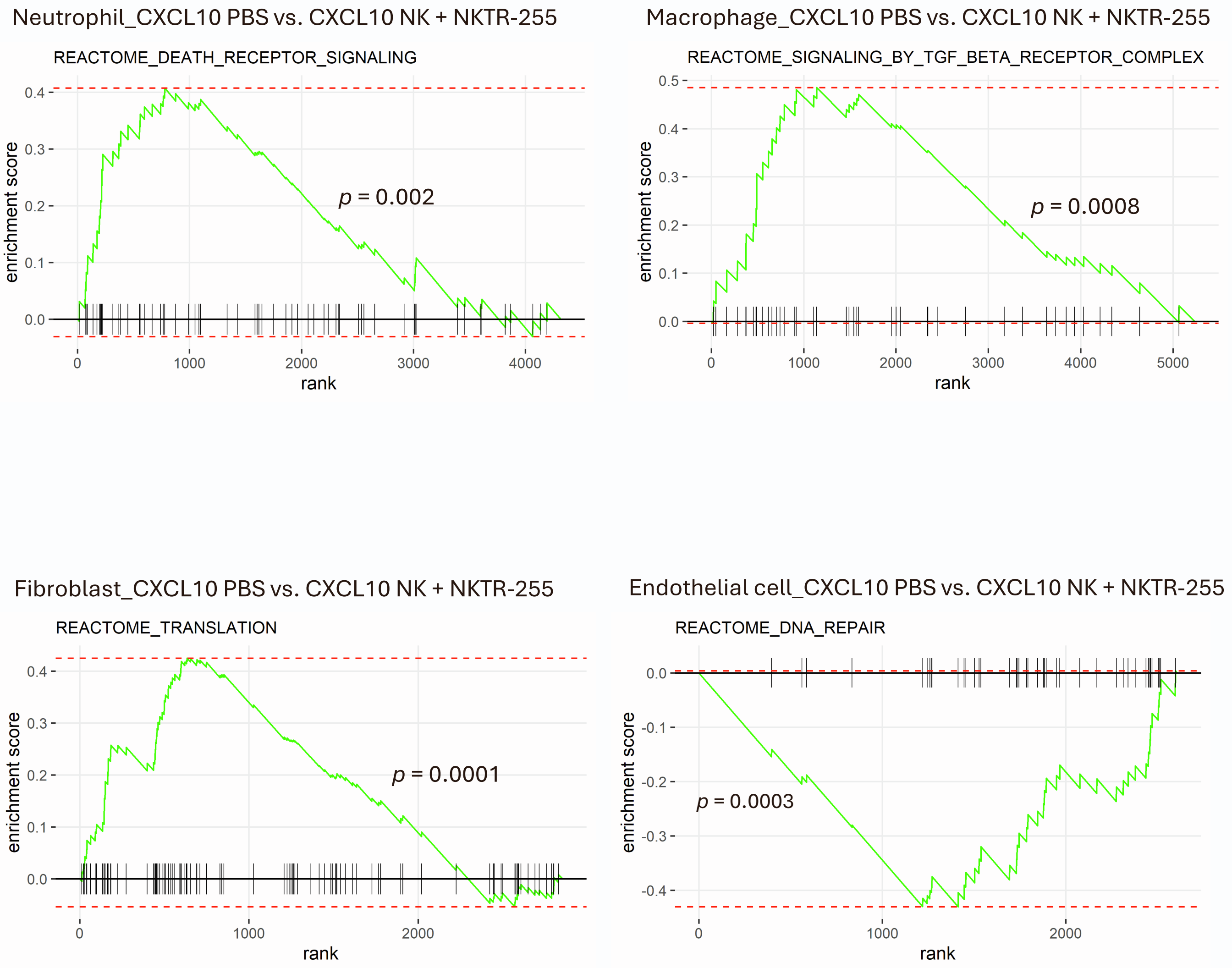

Figure S6. GSEA-enrichment plots of the top mouse gene set enriched in the CXCL10-positive tumor treated with NK cells and NKTR-255 compared with the CXCL10-positive tumor without the treatment

GSEA, gene set enrichment analysis; NK, natural killer; PBS, phosphate buffer solution.

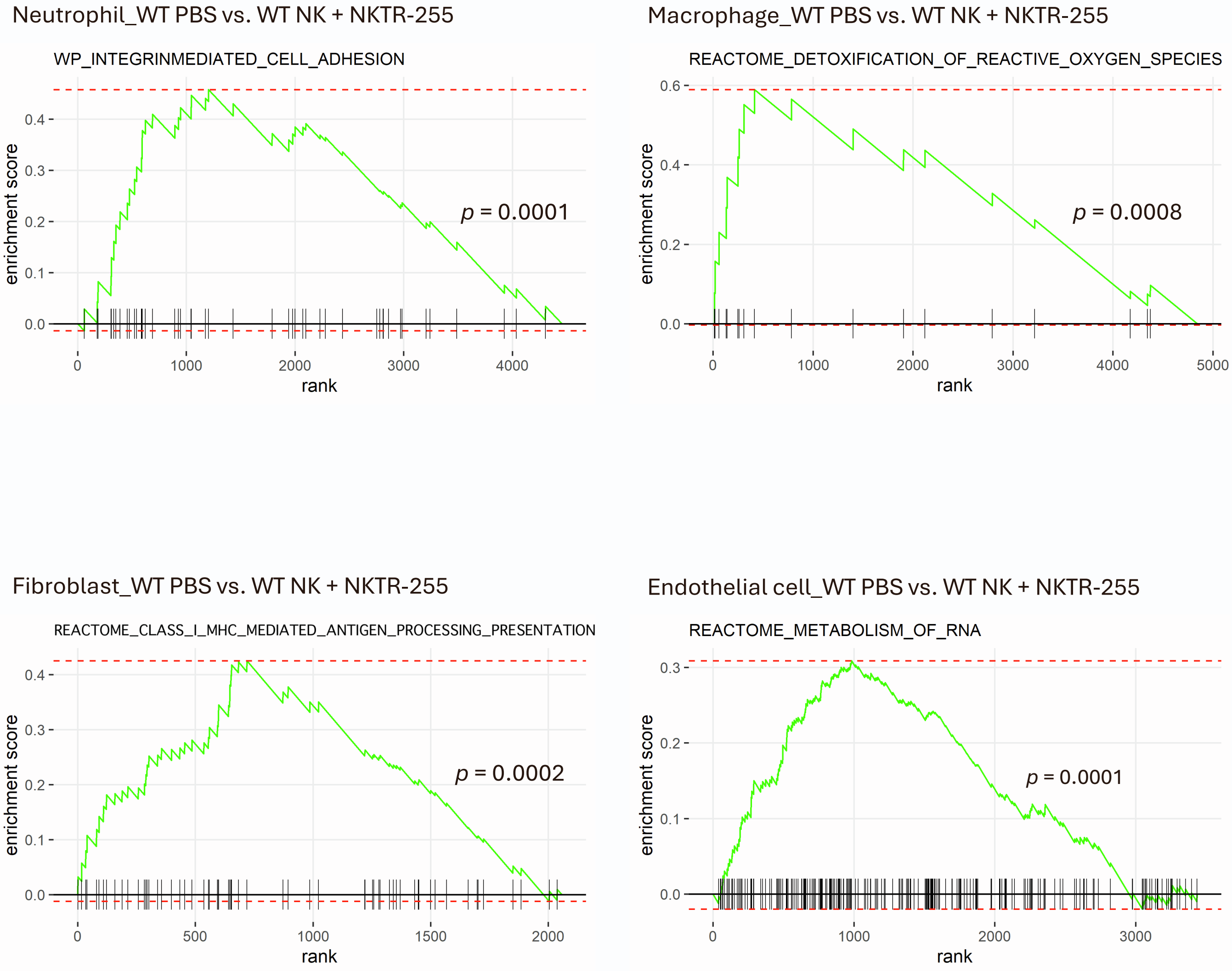

Figure S7. GSEA-enrichment plots of the top mouse gene set enriched in the WT tumor treated with NK cells and NKTR-255 compared with the WT tumor without the treatment

GSEA, gene set enrichment analysis; WT, wild-type; NK, natural killer; PBS, phosphate buffer solution.

SUPPLEMENTAL REFERENCES

1. Finck, R., Simonds, E.F., Jager, A., Krishnaswamy, S., Sachs, K., Fantl, W., Pe'er, D., Nolan, G.P., and Bendall, S.C. (2013). Normalization of mass cytometry data with bead standards. Cytometry A *83*, 483-494. 10.1002/cyto.a.22271.
2. Behbehani, G.K. (2019). Immunophenotyping by Mass Cytometry. Methods Mol Biol *2032*, 31-51. 10.1007/978-1-4939-9650-6_2.
