# Supplementary material for: CXCL10-induced chemotaxis of ex vivo-expanded natural killer cells combined with NKTR-255 enhances anti-tumor efficacy in osteosarcoma
Source: Mol Ther Oncol. 2025 Sep 11;33(4):201051. doi: 10.1016/j.omton.2025.201051 (PMC12509742; doi:10.1016/j.omton.2025.201051)
Supplement: Document S2. Article plus supplemental information [file mmc2.pdf]

Molecular Therapy
Oncology
Original Article

CXCL10-induced chemotaxis of *ex vivo*-expanded natural killer cells combined with NKTR-255 enhances anti-tumor efficacy in osteosarcoma

Shiori Eguchi,[1,2] Wen Luo,[1,3] Hongwen Zhu,[1] Hai M. Hoang,[1] Changxin Xu,[4] Gregory K. Behbehani,[5,6] Kazi L. Tasneem,[1] Janet Ayello,[1] Mario Marcondes,[7] Dean A. Lee,[8] and Mitchell S. Cairo[1,3,9,10]

[1]Department of Pediatrics, New York Medical College, Valhalla, NY 10595, USA; [2]Department of Pediatrics and Developmental Biology, Institute of Science Tokyo, Tokyo 113-8519, Japan; [3]Department of Pathology, Microbiology and Immunology, New York Medical College, Valhalla, NY 10595, USA; [4]James J. Peters Veterans Affairs Medical Center, Bronx, NY 10468, USA; [5]Division of Hematology, The Ohio State University, Columbus, OH 43210, USA; [6]Pelotonia Institute for Immuno-Oncology, The James Comprehensive Cancer Center, The Ohio State University, Columbus, OH 43210, USA; [7]Nektar Therapeutics, San Francisco, CA 94158, USA; [8]Center for Childhood Cancer Research, Abigail Wexner Research Institute at Nationwide Children's Hospital, Columbus, OH 43205, USA; [9]Department of Medicine, New York Medical College, Valhalla, NY 10595, USA; [10]Department of Cell Biology and Anatomy, New York Medical College, Valhalla, NY 10595, USA

Osteosarcoma (OSA) has a dismal prognosis despite surgical resection and multiagent chemotherapy. While adoptive natural killer (NK) cell therapies have been successful in hematological malignancies, the application in solid tumors is challenging due to a tumor microenvironment (TME) that impairs NK cell tumor infiltration. Here, we found that *ex vivo* expansion of NK cells significantly increases the expression of C-X-C motif chemokine receptor 3 (CXCR3), one of the major proteins in the regulation of NK cell chemotaxis. Engineered over-secretion of CXCR3 ligands, C-X-C motif chemokine ligand (CXCL)9, -10, or -11, from OSA cells significantly enhanced expanded NK cell migration toward OSA cells *in vitro* and infiltration into the TME *in vivo*, with the highest NK infiltration rate in CXCL10-secreting tumors. Infusions of expanded NK cells significantly reduced (p = 0.02), and concomitant treatment with an interleukin (IL)-15 agonist NKTR-255 further reduced tumor burden and significantly increased survival in mice bearing CXCL10-secreting tumors compared with those with wild-type tumors (p = 0.02). Single-cell RNA sequencing and mass cytometry revealed upregulated apoptosis and transforming growth factor-β (TGF-β) signaling as the potential mechanisms of response/resistance to NK cell therapy *in vivo*. Our findings highlight potential application of chemokine-enhanced NK tumor infiltration in combination with an IL-15 agonist as a novel approach to effective treatment of OSA.

INTRODUCTION

Osteosarcoma (OSA) is the most common bone sarcoma in children, adolescents, and young adults (CAYA).[1] Despite intensive therapy including neoadjuvant chemotherapy, surgical resection, and adjuvant chemotherapy,[2] patients with relapsed/refractory disease have a dismal prognosis.[3] The Children's Oncology Group reported 5-year overall survival of 17.7% following relapse.[4] Novel treatment strategies are urgently needed.

Immunotherapies including adoptive transfer of T cells and natural killer (NK) cells have recently been investigated.[5] Due to OSA's very low mutational burden with few, if any, neoantigens and infiltration of T cells, T cell efficacy is limited.[6,7] Unlike T cells, NK cells can kill tumor cells without prior sensitization and serve as off-the-shelf allogeneic cell therapy.[8] The density of activated NK cells in the peripheral blood of patients with relapsed OSA correlates with survival following second-line therapies.[9,10] However, CAYA with OSA have reduced peripheral blood NK cells at diagnosis compared with healthy, age-matched controls.[11] Furthermore, NK cell therapies in solid tumors are challenging[12] due to impaired chemotaxis to the tumor microenvironment (TME).[13] Enhancing NK numbers, function, and tumor infiltration may improve anti-OSA efficacy.

To address NK cell scarcity, we developed the antigen-presenting cell line, K562-mbIL21-41BBL, which expanded NK cells 30- to 35,000-fold.[14] Furthermore, we have successfully demonstrated upregulation of activating receptors including NKG2D, NKp30, and NKp44 on expanded NK cells.[15] NK cell persistence and function can be enhanced through exogenous interleukin (IL)-15.[16,17] However, the use of recombinant human IL-15 (rhIL-15) is limited by its rapid plasma clearance.[18] We have been utilizing NKTR-255, a novel polymer-conjugated IL-15 agonist with a longer half-life, which significantly induces pSTAT5 signaling and enhances NK cell proliferation and cytotoxicity.[19,20] We demonstrated that NKTR-255 significantly

Received 25 July 2025; accepted 9 September 2025;
https://doi.org/10.1016/j.omton.2025.201051.

Correspondence: Mitchell S. Cairo, MD, Department of Pediatrics, New York Medical College, Valhalla, NY 10595, USA.
E-mail: mitchell_cairo@nymc.edu

Published by Elsevier Inc. on behalf of The American Society of Gene and Cell Therapy.
This is an open access article under the CC BY license (http://creativecommons.org/licenses/by/4.0/).

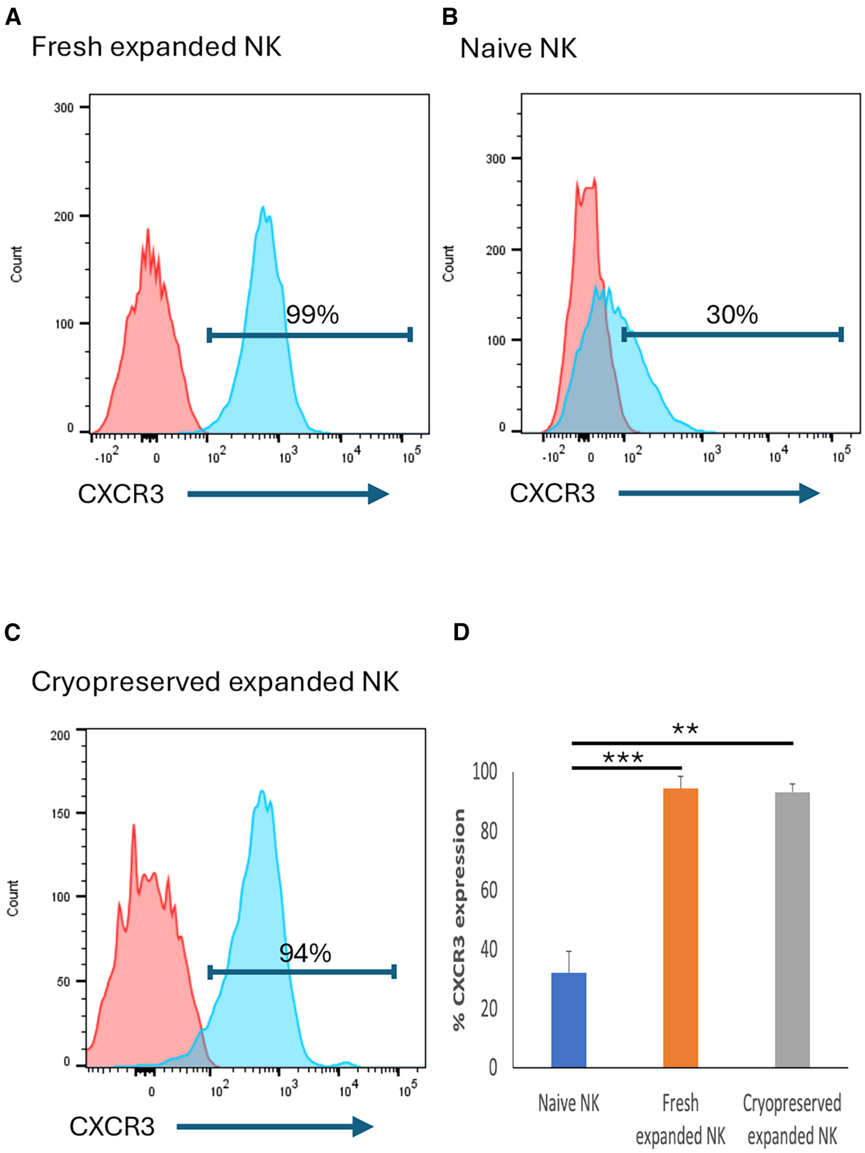

Figure 1. Increased CXCR3 expression on NK cells after *ex vivo* expansion

Histogram of CXCR3 surface expression on (A) fresh NK cells after 15–16 days of co-culture with K562-mbIL21-41BBL feeder cells in the presence of IL-2, (B) naive NK cells directly after isolation from healthy peripheral blood, or (C) cryopreserved NK cells after 14–16 days of co-culture with K562-mbIL21-41BBL feeder cells in the presence of IL-2. The *blue histogram* represents NK cells stained with a PE-Cy7-conjugated anti-CXCR3 antibody. The *red histogram* represents the isotype control. One of the three representative experiments is shown. (D) Comparison of CXCR3 surface expression among naive, fresh expanded, and cryopreserved expanded NK cells. Data are represented as mean ± SD of 3 independent biological replicates using NK cells from 3 different donors. **$p < 0.01$, ***$p < 0.001$ (Student's t test). Expanded NK cells (fresh or cryopreserved) had significantly higher CXCR3 expression compared with naive NK cells. The comparison between fresh and cryopreserved expanded NK cells was not statistically significant. NK, natural killer; IL, interleukin; SD, standard deviation.

enhanced chimeric antigen receptor (CAR) NK cell efficacy against neuroblastoma and Ewing sarcoma *in vitro* and *in vivo*.[21,22]

NK cell migration and infiltration into the TME are in large part controlled by chemokine concentration gradients. One of the major proteins in the regulation of chemotaxis is C-X-C motif chemokine receptor 3 (CXCR3), particularly type A, which directs immune cells toward its ligands including C-X-C motif chemokine ligand (CXCL) 9, -10, or -11.[23,24] In this study, we demonstrate that *ex vivo* expansion significantly upregulated CXCR3 expression on NK cells. We hypothesized that engineering OSA cells to over-secrete CXCL9, -10, and -11 would enhance NK cell chemotaxis to the TME, thereby improving therapeutic efficacy. Furthermore, we investigated whether the novel combination of chemokine-driven recruitment of expanded NK cells and NKTR-255-mediated improvement of NK cell function and persistence could synergistically reduce tumor burden and increase survival of OSA xenografted mice.

RESULTS

Over-secretion of CXCL9, -10, and -11 enhanced expanded NK cell chemotaxis toward OSA cells

We found that CXCR3 expression was significantly higher on *ex vivo*-expanded NK cells (Figure 1A) compared with naive NK cells ($p < 0.001$) (Figure 1B), even after cryopreservation ($p = 0.002$) (Figure 1C). This was observed in NK cells from 3 different donors (Figure 1D). We set out to investigate whether engineered secretion of CXCR3 ligands CXCL9, -10, and -11 would have any effect on expanded NK cell chemotaxis toward OSA cells. After retroviral transduction of OSA cells, we observed significantly increased levels of chemokines secreted from virus-infected 143B cells compared with wild-type (WT) cells (Figure 2A). We observed similar results in U2OS cells (Figure 2B) and luciferase-expressing 143B cells (Figure 2C) and U2OS cells (Figure 2D) that we used for subsequent functional assays. Importantly, in the transwell chemotaxis assay, we observed significantly enhanced migration of expanded NK cells toward the conditioned media from *CXCL9*, *-10*, and *-11* virus-infected 143B and U2OS cells relative to WT cells, both luciferase non-expressing (Figures 3A and 3B) and expressing cells (Figures 3C and 3D). To investigate whether CXCL9, -10, or -11 secretion by OSA cells would induce any change in their sensitivity to NK cells, we performed the *in vitro* luciferase-based cytotoxicity assay. We found that CXCL9, -10, or -11-secreting 143B and U2OS cells were as sensitive

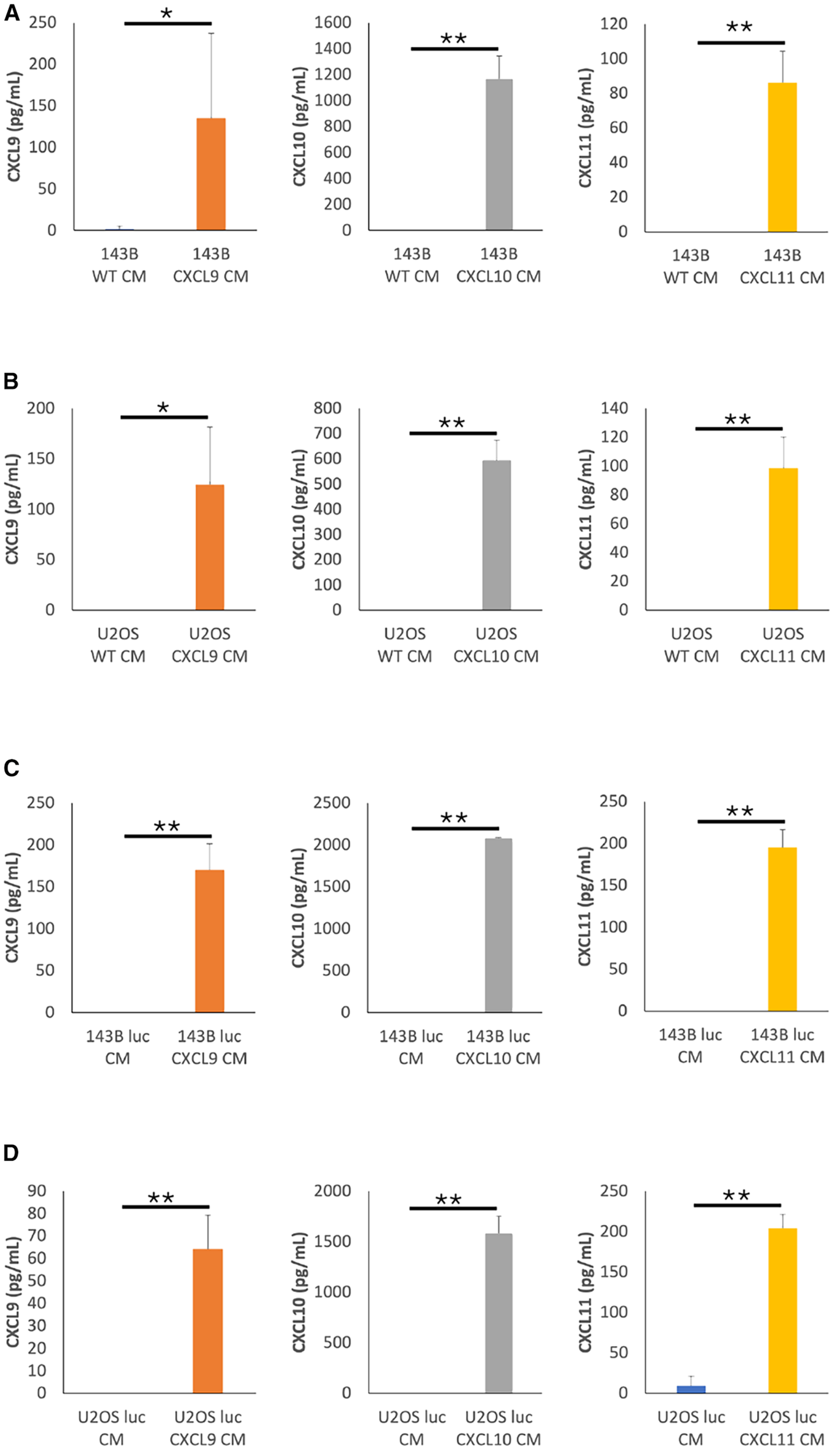

Figure 2. CXCL9, -10, or -11 over-secretion from *CXCL9*, *-10*, or *-11* virus-infected OSA cells
Production of CXCL9, -10, and -11 by (A) *CXCL9*, *-10*, and *-11* virus-infected 143B cells compared with WT 143B cells, (B) *CXCL9*, *-10*, and *-11* virus-infected U2OS cells compared with WT U2OS cells, (C) *CXCL9*, *-10*, and *-11* virus-infected 143B luc cells compared with non-infected 143B luc cells, and (D) *CXCL9*, *-10*, and *-11* virus-infected U2OS luc cells compared with non-infected U2OS luc cells. CM from 143B or U2OS (1.25×10^6 cells/mL) were collected after 6 h of incubation and analyzed by the ELISA. Experiments were performed using duplicate samples to obtain an average value. Three to 4 biological repeat experiments were performed. Data are represented as mean ± SD of 3–4 independent experiments. *$p < 0.05$, **$p < 0.01$ (Student's t test) in (A), (B), (C), and (D). CM from *CXCL9*, *-10*, or *-11* virus-infected OSA cells (143B, U2OS, 143B luc, or U2OS luc) secreted significantly increased levels of chemokines compared with their non-infected counterparts. OSA, osteosarcoma; WT, wild-type; luc, luciferase-expressing; CM, conditioned media; ELISA, enzyme-linked immunosorbent assay; SD, standard deviation.

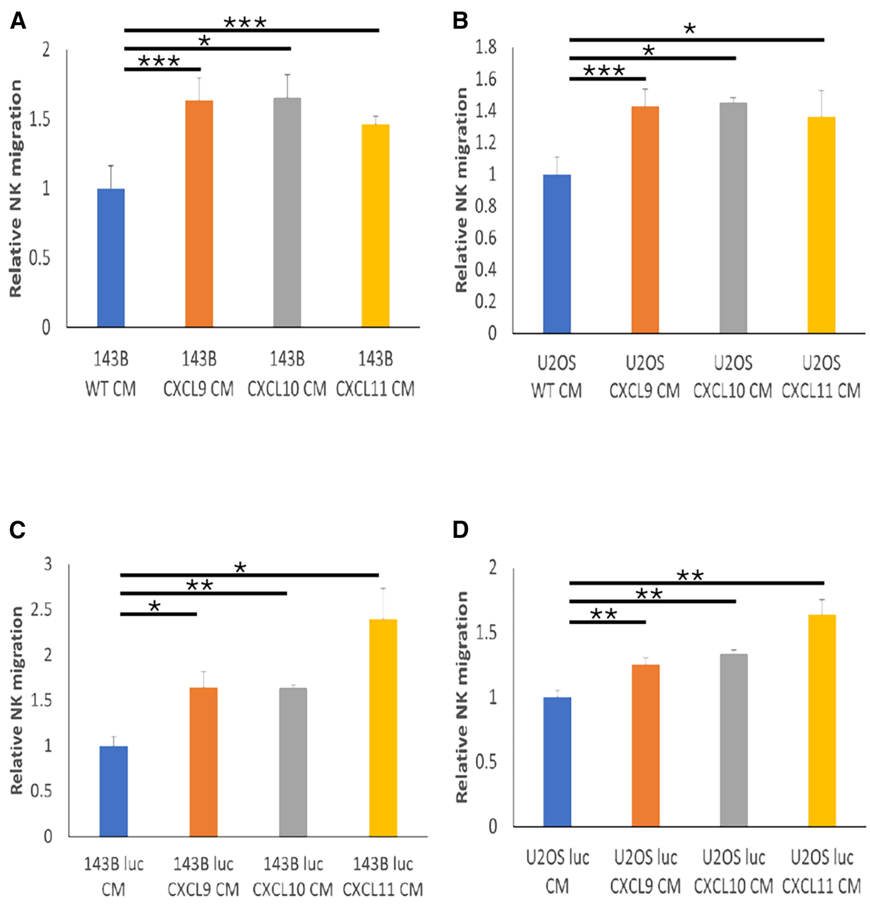

Figure 3. Enhanced *in vitro* NK migration toward *CXCL9*, *-10*, or *-11* virus-infected OSA CM

Transwell migration assay of expanded NK cells toward (A) CM from *CXCL9*, *-10*, and *-11* virus-infected 143B cells relative to WT 143B CM, (B) CM from *CXCL9*, *-10*, and *-11* virus-infected U2OS cells relative to WT U2OS CM, (C) CM from *CXCL9*, *-10*, and *-11* virus-infected 143B luc cells relative to non-infected 143B luc CM, and (D) CM from *CXCL9*, *-10*, and *-11* virus-infected U2OS luc cells relative to non-infected U2OS luc CM. Serum-free media were cultured with 143B or U2OS cells (1.25×10^6 cells/mL) for 6 h and transferred to 24-well plates. Expanded NK cells were added at 5×10^5 per transwell insert (5-μm pore size filter) and incubated for 2 h. Data are represented as mean ± SD of 3–4 independent biological replicates. *$p < 0.05$, **$p < 0.01$, ***$p < 0.001$ (Student's t test) in (A), (B), (C), and (D). CM from *CXCL9*, *-10*, or *-11* virus-infected OSA cells (143B, U2OS, 143B luc, or U2OS luc) significantly enhanced NK migration compared with CM from their non-infected counterparts. NK, natural killer; OSA, osteosarcoma; CM, conditioned media; WT, wild-type; luc, luciferase-expressing; SD, standard deviation.

as, if not more sensitive than, WT cells to NK cells at different effector-to-target (E:T) ratios (Figures 4A and 4B).

Increased chemotaxis and intratumoral infiltration of adoptively transferred expanded NK cells in CXCL9, -10, or -11-positive OSA tumors *in vivo*

We next investigated whether expanded NK cells would migrate better toward CXCL9, -10, or -11-positive than WT OSA xenograft tumors *in vivo*. NOD/SCID/gamma$^{-/-}$ (NSG) mice were inoculated subcutaneously (s.c.) with both WT and chemokine (either CXCL9, -10, or -11)-secreting 143B cells on separate flanks (left: WT; right: CXCL9, -10, or -11). Tumor-bearing mice were injected intraperitoneally (i.p.) with luciferase-expressing expanded NK cells (Figure 5A). Bioluminescence imaging of the mice showed significantly enhanced NK migration toward CXCL9, -10, or -11-positive tumors relative to WT tumors (p = 0.03, p = 0.02, and p = 0.005, respectively) (Figures 5B and 5C). Of note, tumor sizes were similar on the left and right (Figures S1A and S1B). To evaluate NK cell tumor infiltration, we i.p. injected expanded NK cells together with NKTR-255, which was used to enhance NK cell persistence, 5–6 days after tumor inoculation and harvested tumors 5–7 days after NK cell injection (Figure 5D). Flow cytometry of dissociated tumors showed significantly increased numbers of human CD45$^+$ cells in CXCL9, -10, or -11-positive tumors relative to WT tumors (p = 0.01, p = 0.03, and p = 0.04, respectively) (Figure 5E), demonstrating significantly enhanced NK cell infiltration into the chemokine-secreting tumors. Notably, significantly more NK cells infiltrated CXCL10-positive (CXCL10$^+$) tumors than CXCL9 or -11-positive tumors (p = 0.04 and p = 0.03, respectively) (Figure 5E). We therefore focused on CXCL10 in the subsequent studies. Further evaluation with immunohistochemical staining to localize the infiltrated NK cells in the tumors was not sensitive enough to detect NK cells (data not shown).

Adoptive transfer of expanded NK cells with NKTR-255 significantly reduced tumor burden and increased survival in mice bearing CXCL10$^+$ tumors compared with WT tumors

In addition to treatment with NK cells alone, we tested the combinatorial therapy of NK cells and NKTR-255 to further improve the anti-OSA efficacy of NK cells. *In vitro*, NKTR-255 significantly enhanced NK cytotoxicity against both WT and CXCL10$^+$ 143B cells compared with NK cells alone at different E:T ratios (Figure 6A). To investigate whether WT and CXCL10$^+$ tumor cells would grow similarly in tissue culture, we evaluated proliferation of these cells with or without luciferase expression. The growth curves showed that CXCL10$^+$ 143B cells without luciferase expression grew similarly to their WT counterparts (Figure 6B). However, luciferase-expressing CXCL10$^+$ 143B cells grew significantly differently from luciferase-expressing WT 143B cells (p = 0.02) (Figure S2A). Furthermore, luciferase-expressing CXCL10$^+$ 143B cells had significantly higher luciferase activity compared with luciferase-expressing WT 143B cells (p = 0.02) (Figure S2B). We therefore utilized the 143B cells without

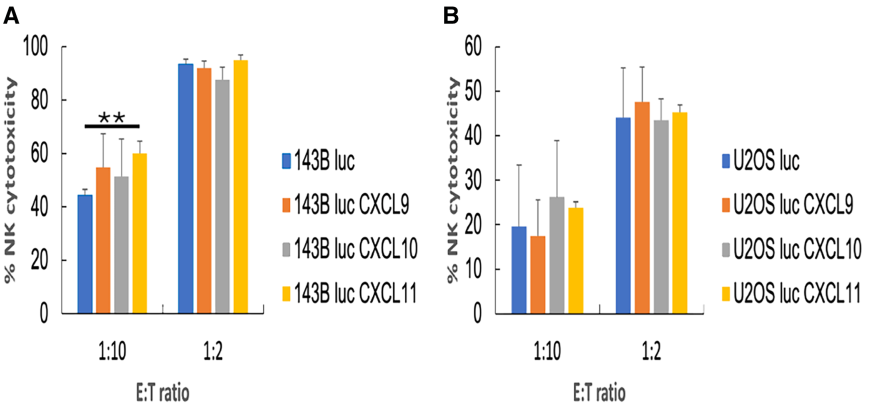

Figure 4. Maintained sensitivity of CXCL9, -10, or -11-secreting OSA cells to NK cell killing

NK cytotoxicity against (A) *CXCL9*, *-10*, and *-11* virus-infected and non-infected 143B luc cells, and (B) *CXCL9*, *-10*, and *-11* virus-infected and non-infected U2OS luc cells. Data are represented as mean ± SD of triplicates in a representative experiment. Experiments were repeated 3 times with similar results. $^{**}p < 0.01$ (Student's t test) in (A). *CXCL11* virus-infected 143B luc cells were significantly more sensitive to NK cells than non-infected 143B luc cells at an E:T ratio = 1:10. The other comparison between *CXCL9*, *-10*, or *-11* virus-infected and non-infected OSA cells (143B luc or U2OS luc) at E:T ratios = 1:10 and 1:2 was not statistically significant. OSA, osteosarcoma; NK, natural killer; luc, luciferase-expressing; SD, standard deviation; E:T, effector-to-target.

luciferase expression for the subsequent *in vivo* study. For *in vivo* study, NSG mice were inoculated s.c. with either WT or CXCL10^{+} 143B cells and injected i.p. with phosphate-buffered saline (PBS), NKTR-255, NK, or NK+NKTR-255 (Figure 6C). Caliper measurements of the tumors revealed that, while WT and CXCL10^{+} tumor burden was similar with PBS treatment (p = 0.65) (Figure 6D), NK cell treatment significantly reduced tumor burden in mice bearing CXCL10^{+} tumors compared with WT tumors (p = 0.02) (Figure 6D). However, NK cell treatment alone did not significantly improved survival in mice bearing CXCL10^{+} tumors compared with WT tumors (median survival 37 vs. 35 days, p = 0.13) (Figure 6E). NK cell treatment, when combined with NKTR-255, significantly reduced CXCL10^{+} tumor burden compared with PBS, NKTR-255 alone, or NK alone ($p < 0.001$, $p < 0.001$, and p = 0.02, respectively) (Figure 6D), while we did not observe a significant difference in WT tumor burden among the treatment groups (Figure 6D). The combinatorial therapy of NK cells and NKTR-255 had a more significant effect on reducing tumor burden in mice bearing CXCL10^{+} tumors than WT tumors (p = 0.03) (Figure 6D). Furthermore, while WT 143B-engrafted mice had similar survival regardless of the treatment groups (Figure 6E), CXCL10^{+} 143B-engrafted mice treated with NK+NKTR-255 had significantly increased survival compared with those treated with PBS or NKTR-255 alone (p = 0.03 and p = 0.008, respectively) (Figure 6E). In addition, NK+NKTR-255 significantly prolonged survival of mice bearing CXCL10^{+} tumors compared with mice bearing WT tumors (median survival 41 vs. 35 days, p = 0.02) (Figure 6E).

Identification of mechanisms of response/resistance to the NK combinatorial therapy against OSA tumors *in vivo*

NSG mice were inoculated s.c. with both WT and CXCL10^{+} 143B cells on separate flanks (left: WT; right: CXCL10). One day following tumor inoculation, expanded NK cells were injected i.p. with NKTR-255, followed by once-a-week NK cell injection. Mice not injected with NK cells and NKTR-255 were injected i.p. with PBS. Tumors were collected 21–23 days following tumor injection and dissociated into single cells (Figure 7A) for single-cell RNA sequencing (scRNA-seq) and mass cytometry. Dimensional reduction via uniform manifold approximation and projection separated the human cells into 10 clusters (Figure S3A) and the mouse cells into 4 clusters (Figure S3B). The 10 human clusters were identified as OSA cells, and the 4 mouse clusters were identified as neutrophils, macrophages, fibroblasts, and endothelial cells (see materials and methods, scRNA-seq) (Figure S3B). More mouse leukocytes, specifically neutrophils, were detected in CXCL10^{+} tumors relative to WT tumors (Figure 7B). Increased mouse leukocyte infiltration into CXCL10^{+} tumors was confirmed by mass cytometry (Figure 7C), although identification of the subpopulation was difficult due to the small number of infiltrated cells. Neither scRNA-seq nor mass cytometry detected human leukocytes. This suggests that, although CXCL10 induced significantly enhanced migration and infiltration of NK cells, the number of infiltrated NK cells relative to the tumor volume was too small to be detected by scRNA-seq or mass cytometry. Interestingly, we found that an apoptotic gene *IFIT2* was upregulated in NK+NKTR-255-treated compared to PBS-treated OSA cells (Figure 7D). In addition, we observed a higher percentage of mitochondrial genes expressed in NK+NKTR-255-treated OSA cells (Figure 7E), indicating a higher level of apoptosis in these cells. We next performed gene set enrichment analysis (GSEA) across major human cell populations to reveal enrichment of pathways in these cells. Phosphatidylinositol 3-kinase-AKT signaling, CXCL10 downstream signaling, was significantly upregulated in the PBS-treated CXCL10^{+} tumor compared with the PBS-treated WT tumor in the human cluster 8 (p = 0.001). We identified the enrichment of pathways related to cell death in NK+NKTR-255-treated compared with PBS-treated CXCL10^{+} tumor cells (Figures 7F and S4) but barely in WT tumor cells (Figure S5). Consistently, increased expression of an apoptotic marker cPARP in NK+NKTR-255-treated CXCL10^{+} tumors was shown by mass cytometry (p = 0.03) (Figures 7G and 7H). In addition, we performed GSEA on mouse cells (Figures S6 and S7), and the top positively enriched pathway was transforming growth factor (TGF)-β receptor complex in macrophages (identified as described in materials and methods, scRNA-seq) infiltrated in the NK+NKTR255-treated CXCL10^{+} tumor compared with the PBS-treated CXCL10^{+} tumor ($p < 0.001$) (Figure 7I).

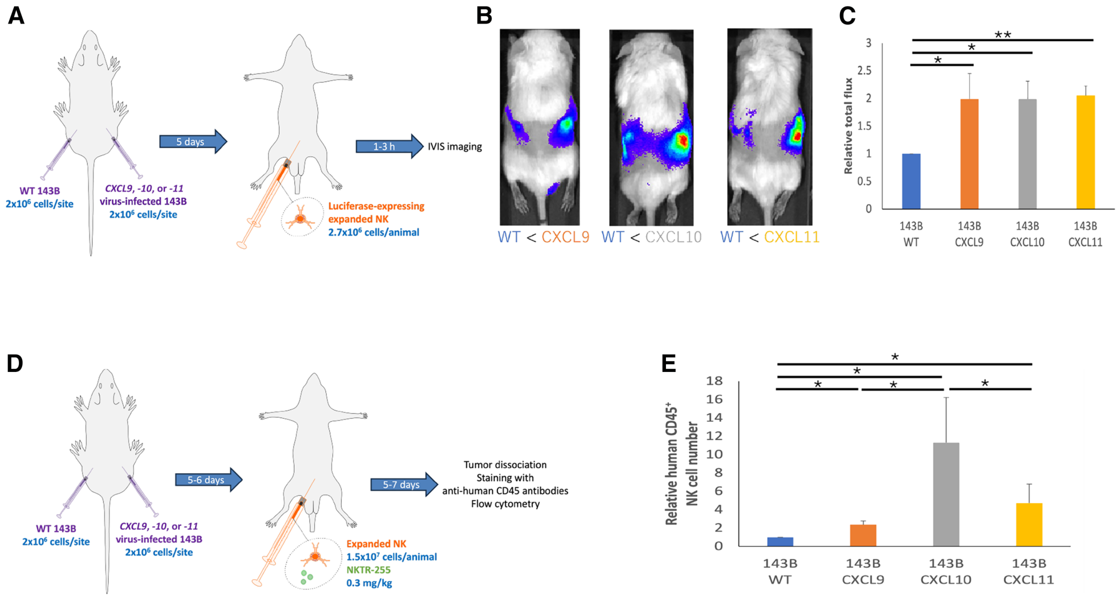

Figure 5. Increased *in vivo* NK chemotaxis to *CXCL9*, *-10*, or *-11* virus-infected OSA

NSG mice were inoculated s.c. with 2×10^6 WT and chemokine (either CXCL9, -10, or -11)-secreting 143B cells in each flank of the same mouse. (A) Xenograft model for migration experiments: five days following tumor inoculation (when tumors were palpable), luciferase-expressing NK cells were injected (i.p. 2.7×10^6). Mice were imaged using bioluminescent imaging 1–3 h following NK cell injection ($n = 3$ mice/group). (B) Example of *in vivo* imaging of a mouse inoculated with WT (left flank) and chemokine (either CXCL9, -10, or -11)-secreting 143B cells (right flank) treated as described in (A). One of the three representative mice/group is shown. (C) Luminescence from chemokine (either CXCL9, -10, or -11)-secreting tumors relative to WT tumors. Data are represented as mean ± SD. $^*p < 0.05$, $^{**}p < 0.01$ (Student's t test). *CXCL9*, *-10*, or *-11* virus-infected 143B tumors significantly enhanced NK migration compared with non-infected 143B tumors. The other comparison was not statistically significant. (D) Xenograft model for infiltration experiments: 5–6 days following tumor inoculation, NK cells were injected (i.p. 1.5×10^7) with NKTR-255 (i.p. 0.3 mg/kg). Tumors were collected 5–7 days after NK cell injection, dissociated, stained with an anti-human CD45 antibody, and analyzed on a flow cytometer ($n = 3$ mice/group). (E) Numbers of human CD45$^+$ cells in dissociated CXCL9, -10, or -11-positive tumors relative to WT tumors. Data are represented as mean ± SD. $^*p < 0.05$ (Student's t test). *CXCL9*, *-10*, or *-11* virus-infected 143B tumors significantly enhanced NK infiltration compared with non-infected 143B tumors. *CXCL10* virus-infected 143B tumors significantly enhanced NK infiltration compared with *CXCL9* or *-11* virus-infected 143B tumors. The comparison between *CXCL9* virus-infected and *CXCL11* virus-infected 143B tumors was not statistically significant. NK, natural killer; OSA, osteosarcoma; NSG, NOD/SCID/gamma$^{-/-}$; s.c., subcutaneously; WT, wild-type; i.p., intraperitoneally; SD, standard deviation.

DISCUSSION

In the current study, we demonstrated that CXCR3 is highly expressed on *ex vivo*-expanded human NK cells. Expanded NK cells exhibited significantly enhanced chemotaxis toward OSA secreting CXCL9, -10, and -11 in two different OSA cell lines and *in vivo*. Adoptive transfer of these NK cells significantly reduced tumor burden in mice bearing CXCL10$^+$ OSA tumors. Concomitant treatment with NKTR-255 further significantly suppressed tumor progression and significantly prolonged survival in mice bearing CXCL10$^+$ OSA tumors. While apoptosis was upregulated in CXCL10$^+$ OSA tumors following treatment with NK cells and NKTR-255, we also observed increased TGF-β signaling, implying a resistance mechanism.

Despite its efficacy in hematological malignancies, adoptive NK cell therapy has yet to demonstrate beneficial clinical responses in solid tumors,[25–28] partly due to poor NK cell trafficking to the TME.[13] Efforts have been made to improve NK cell chemotaxis by genetically modifying NK cells to express chemokine receptors to chemokines highly secreted by tumors.[29] However, this approach often results in low receptor expression and compromised NK cell viability. In contrast, our study showed that CXCR3 was readily expressed on more than 90% of NK cells following *ex vivo* expansion. The observed CXCR3 upregulation is likely attributable to the combined action of IL-21, provided by K562-mbIL21-41BBL feeder cells, and IL-2. Both cytokines are known to engage the JAK-STAT axis, culminating in the induction of critical transcriptional regulators such as T-bet,[30,31] which plays a central role in regulating CXCR3 expression on NK cells.[32] Notably, this upregulation occurred without genetic modification, which brings an advantage that we can use active NK cells without impairing viability. The high CXCR3 expression also persisted after cryopreservation, facilitating clinical applicability. There are two isoforms of CXCR3, A and B, with distinct functions. CXCR3A promotes immune cell chemotaxis in response to ligands CXCL9, -10, and -11, while CXCR3B mediates growth inhibition, apoptosis, and anti-migratory/anti-angiogenic effects.[24] The two isoforms can be expressed on the same cell. However, it was reported that CXCR3A is the predominant type expressed on activated NK cells.[33,34] Furthermore, our data showed significantly enhanced NK cell chemotaxis to CXCL9, -10, and -11-secreting

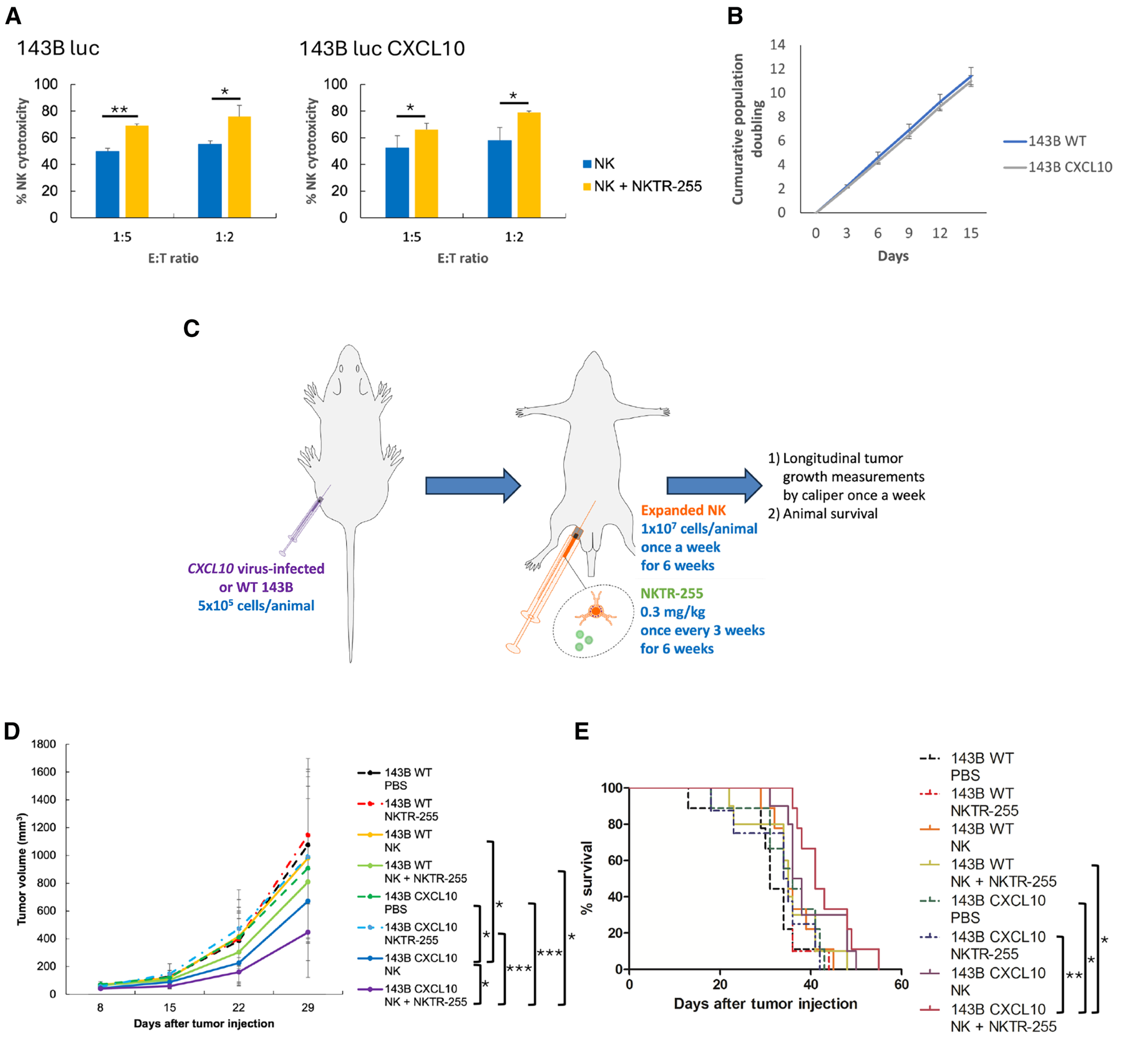

Figure 6. Decreased tumor burden and prolonged survival in mice bearing CXCL10⁺ tumors following adoptive transfer of NK cells with NKTR-255

(A) NK cytotoxicity against *CXCL10* virus-infected and non-infected 143B luc cells with or without NKTR-255 (40 ng/mL). Data are represented as mean ± SD of the triplicates in a representative experiment. Experiments were repeated 3 times with similar results. $^{*}p < 0.05$, $^{**}p < 0.01$ (Student's t test). NKTR-255 significantly enhanced NK cytotoxicity against both *CXCL10* virus-infected and non-infected 143B luc cells compared with NK cells alone at E:T ratios = 1:5 and 1:2. (B) Growth curve of WT and CXCL10^{+} 143B cells. Data are represented as mean ± SD of 3 independent biological replicates. The comparison between WT and CXCL10^{+} 143B cells was not statistically significant (Student's t test). (C) Xenograft model for tumor progression and survival analysis: NSG mice were inoculated s.c. with 5×10^5 WT or CXCL10^{+} 143B cells in individual mice. Mice were divided into 4 treatment groups: PBS, NKTR-255, NK, and NK+NKTR-255. NK cell injections (i.p. 1×10^7) and NKTR-255 injections (i.p. 0.3 mg/kg) started 1 day following tumor inoculation. A total of six infusions of NK cells were administered every 7 days. A total of two infusions of NKTR-255 were administered every 3 weeks (n = 8–10 mice/group). (D) Tumor burden measured by a digital caliper 8–29 days after tumor inoculation in mice bearing either WT or CXCL10^{+} tumors treated with PBS, NKTR-255 alone, NK cells alone, or the combinatorial therapy of NK cells and NKTR-255. Tumor volume at the time of death was used for subsequent tumor volume of mice that died before 29 days post tumor inoculation. n = 8–10 mice/group. Data are represented as mean ± SD. $^{*}p < 0.05$, $^{***}p < 0.001$ (ANOVA with post hoc Bonferroni test). NK significantly reduced CXCL10^{+} tumor burden compared with WT tumor burden. NK significantly reduced CXCL10^{+} tumor burden compared with PBS. NK+NKTR-255 significantly reduced CXCL10^{+} tumor burden compared with WT tumor burden. NK+NKTR-255 significantly reduced CXCL10^{+} tumor burden compared with PBS, NKTR-255, or NK. The comparison of WT and CXCL10^{+} tumor burden treated with PBS or NKTR-255 was not statistically significant. The comparison of WT tumor burden among the treatment groups was not statistically significant. (E) Survival of mice bearing either WT or CXCL10^{+} 143B tumors treated with PBS, NKTR-255 alone, NK cells alone, or the combinatorial therapy of NK cells and NKTR-255. n = 8–10 mice/group. $^{*}p < 0.05$, $^{**}p < 0.01$ (log rank test). NK+NKTR-255 significantly extended survival of mice bearing CXCL10^{+} tumors compared with mice bearing WT tumors. NK+NKTR255 significantly extended survival of mice bearing CXCL10^{+} tumors compared with PBS or NKTR-255 alone. The comparison of survival of mice bearing WT or CXCL10^{+} tumors treated with PBS or NKTR-255 was not statistically significant. The comparison of survival of mice bearing WT tumors among the treatment groups was not statistically significant. NK, natural killer; luc, luciferase-expressing; SD, standard deviation; WT, wild-type; NSG, NOD/SCID/gamma$^{-/-}$; s.c.; subcutaneously; PBS, phosphate buffer solution; i.p., intraperitoneally; ANOVA, analysis of variance.

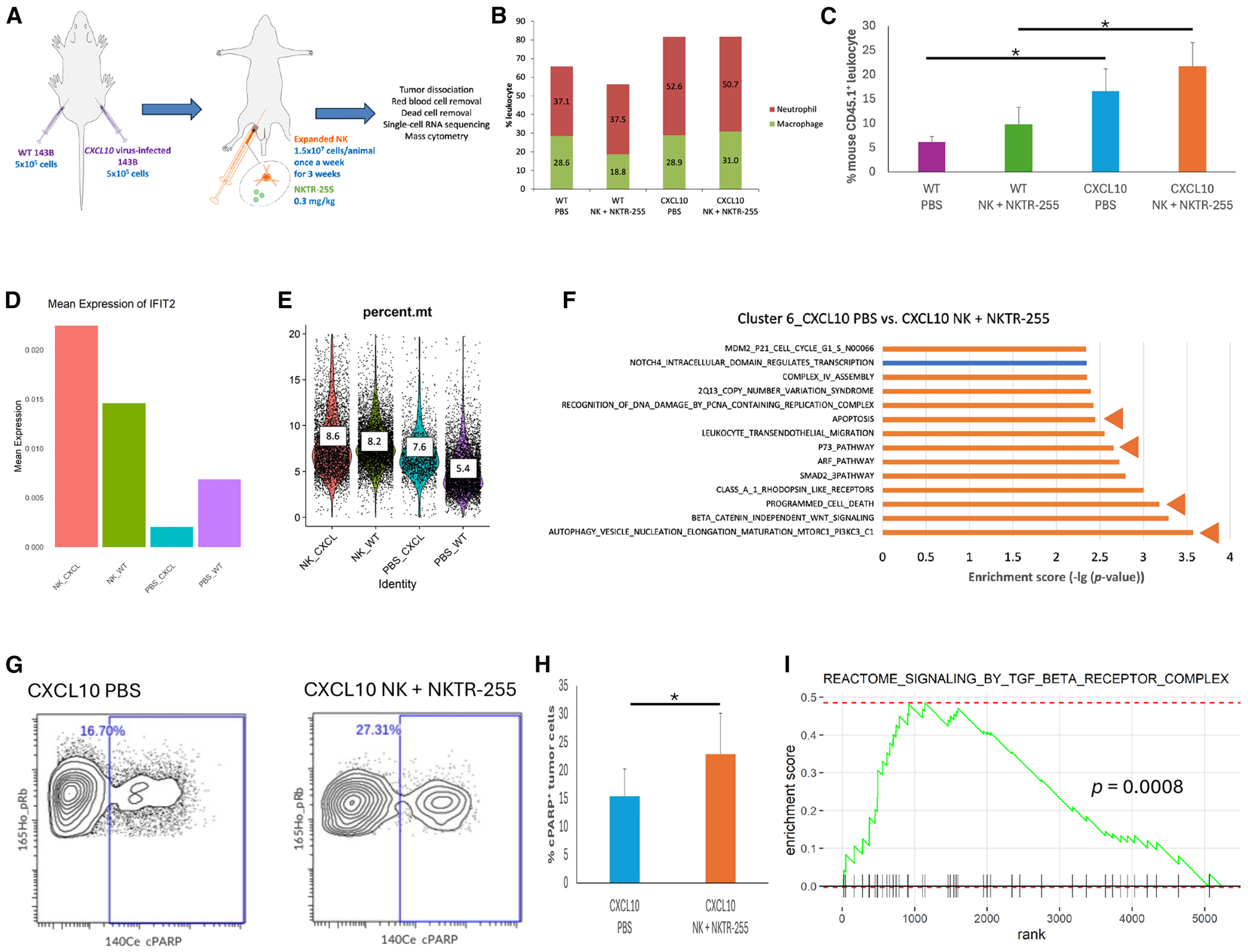

Figure 7. Upregulation of apoptosis and TGF-β signaling following treatment with NK cells and NKTR-255

(A) Xenograft model for analysis of response and resistance mechanisms: NSG mice were inoculated s.c. with 5×10^5 WT and CXCL10$^+$ 143B cells in each flank of the same mouse. Mice were divided into 2 treatment groups: PBS and NK+NKTR-255. NK cell injections (i.p. 1.5×10^7) and NKTR-255 injections (i.p. 0.3 mg/kg) started 1 day following tumor inoculation. A total of three infusions of NK cells were administered every 7 days. Tumors were collected 21–23 days after tumor injection, dissociated, and analyzed by scRNA-seq (n = 1 mouse/group) and mass cytometry (n = 3 mice/group) following red blood cell removal and dead cell removal. (B) Percentage of total leukocytes, neutrophils, and macrophages (identified by scRNA-seq as described in materials and methods) in mouse cells infiltrating into each tumor. (C) Percentage of mouse CD45.1$^+$ cells (detected by mass cytometry) in total live cells in each tumor. Data are represented as mean ± SD. *$p < 0.05$ (Student's t test). PBS-treated CXCL10$^+$ tumors significantly enhanced mouse leukocyte infiltration compared with PBS-treated WT tumors. NK+NKTR-255-treated CXCL10$^+$ tumors significantly enhanced mouse leukocyte infiltration compared with NK+NKTR-255-treated WT tumors. (D) Mean expression of *IFIT2* by pooled analysis of scRNA-seq data on OSA cells in each tumor. (E) Violin plot detailing the percentage of mitochondrial genes in cells that passed QC thresholds (percentage of mitochondrial genes <20%, nCount_RNA <25,000, nFeature_RNA >200 and <5,000) from each tumor. Box represents median. (F) GSEA bar chart showing enrichment scores for the hallmark pathways that were different between CXCL10$^+$ tumors with and without treatment with NK cells and NKTR-255. Pathways upregulated in the CXCL10$^+$ tumor treated with NK cells and NKTR-255 were shown in orange. Pathways downregulated in the CXCL10$^+$ tumor treated with NK cells and NKTR-255 were shown in blue. Pathways indicated by arrowhead (◀) were related to cell death. See also Figure S4. (G) Mass cytometry plot showing pRb vs. cPARP channel of OSA cells (hCD45$^-$mCD45.1$^-$pRb^{high}GAPDH^{high}) in a CXCL10$^+$ tumor with or without treatment with NK cells and NKTR-255. One of the three representative plots/treatment group is shown. (H) Percentage of cPARP$^+$ OSA cells (hCD45$^-$mCD45.1$^-$pRb^{high}GAPDH^{high}) in CXCL10$^+$ tumors with or without treatment with NK cells and NKTR-255. Data are represented as mean ± SD. *$p < 0.05$ (Student's t test). NK+NKTR-255 significantly increased expression of cPARP in CXCL10$^+$ tumors compared with PBS. (I) GSEA enrichment plot on mouse macrophages identified by scRNA-seq. Signaling by TGF-β receptor complex was the top pathway positively enriched in the NK+NKTR-255-treated CXCL10$^+$ tumor compared with the PBS-treated CXCL10$^+$ tumor. See also Figure S6. NK_CXCL, NK+NKTR-255-treated CXCL10$^+$ OSA; NK_WT, NK+NKTR-255-treated WT OSA; PBS_CXCL, PBS-treated CXCL10$^+$ OSA; PBS_WT, PBS-treated WT OSA; NK, natural killer; OSA, osteosarcoma; WT, wild-type; PBS, phosphate buffer solution; NSG, NOD/SCID/gamma$^{-/-}$; s.c., subcutaneously; i.p., intraperitoneally; scRNA-seq, single-cell RNA sequencing; SD, standard deviation; QC, quality control; GSEA, gene set enrichment analysis.

OSA tumors, suggesting prevalent expression of CXCR3A on expanded NK cells.

To our knowledge, in any tumor types, this is the first study demonstrating *in vivo* chemoattraction of NK cells by all three CXCR3 ligands. Among these, CXCL10 induced the strongest NK cell infiltration, which led us to focus on CXCL10 for tumor progression and survival analysis. However, the highest infiltration rate in $CXCL10^+$ tumors may be due to higher CXCL10 secretion induced by viral transduction compared with CXCL9 or -11. Optimized CXCL9 or -11 secretion from tumor cells may similarly improve NK cell recruitment. A previous study detected CXCL10 in the majority of patient-derived OSA using a membrane-based antibody array,[35] but the levels may be insufficient to attract NK cells given the low NK cell infiltration in the OSA TME consistently reported in the literature.[36] Establishing chemokine thresholds could help use CXCL9, -10, and -11 as biomarkers to identify patients likely to benefit from adoptive transfer of *ex vivo*-expanded NK cells. For tumors with low endogenous secretion, a strategy to induce over-secretion of these chemokines may improve therapeutic outcomes. Notably, CXCL11 binds a distinct domain on CXCR3 compared with CXCL9 and -10,[37] suggesting that combined over-secretion of CXCL9 and -11, or CXCL10 and -11 may further enhance NK chemotaxis beyond individual ligand effects.

This study also provides the first evidence that NKTR-255 enhances anti-OSA efficacy of *ex vivo*-expanded NK cells. NKTR-255 significantly improved NK cytotoxicity *in vitro* and, when combined with CXCL10-driven NK cell recruitment, led to further reduced tumor burden and prolonged survival *in vivo*. The favorable tolerability of NKTR-255 in our xenograft model, coupled with its extended half-life compared with rhIL-15, supports its clinical potential.[19]

Our transcriptomic and immunologic analysis revealed mechanisms of response and resistance to the combinatorial therapy. Apoptotic gene expression was the highest in NK+NKTR-255-treated $CXCL10^+$ OSA cells, suggesting that NK cells induce apoptosis of OSA cells and the extent of apoptosis correlates with NK cell infiltration in the TME. Future studies incorporating detailed histopathological assessments including necrosis, apoptotic bodies, mitotic activity, and cellular atypia are warranted to verify this mechanism of response. Conversely, increased TGF-β signaling, a known inhibitor of NK cytotoxicity even under IL-15 stimulation,[38] was also observed in the NK+NKTR-255-treated $CXCL10^+$ OSA TME. Future work should explore approaches to counteract TGF-β-mediated resistance in the context of NK+NKTR-255 therapy, such as TGF-β imprinting of NK cells, which we have previously demonstrated can circumvent several mechanisms of TGF-β1-mediated resistance.[39]

This study has a few limitations. First, we used NSG mice, which lack T, B, and NK cells and have impaired macrophage and dendritic cell function.[40] While this allowed us to isolate NK cell-specific effects, NSG mice, even with NKTR-255, lack the homeostatic and survival signals required to maintain adoptively transferred NK cells long enough for meaningful assessment of long-term immune responses. Moreover, we were not able to evaluate the broader immune interactions in the TME. In an immune-competent setting, CXCL9, -10, or -11 may recruit additional CXCR3-expressing endogenous immune cells, such as $CD4^+$ T cells including regulatory T cells (Tregs), $CD8^+$ T cells, subsets of B cells, dendritic cells, and macrophages.[34] IL-15 has been reported to reduce Treg-mediated suppression[41] and promote M1 macrophage polarization,[42] suggesting that NKTR-255 may shift the immune balance in favor of tumor control. Evaluation of these aspects in an immune-competent model is warranted. Second, viral transduction was used solely as a proof of concept and is not clinically applicable. Future efforts will focus on developing an oncolytic virus encoding CXCL9, -10, or -11. Various oncolytic viruses, such as adenovirus, herpes simplex virus, and measles virus, have demonstrated efficacy in preclinical OSA models.[43] We previously reported that an oncolytic herpes simplex virus encoding IL-21 (C021) induced IL-21 secretion from neuroblastoma cells and, when combined with anti-ROR1 CAR NK cells, extended survival in xenografted NSG mice.[44] Similarly, engineering an oncolytic virus to encode CXCL9, -10, or -11 could promote their secretion from OSA cells. Third, the risk of metastasis from CXCL10 over-secretion and the potential of NK+NKTR-255 therapy to inhibit metastatic spread were not evaluated in this study. Significant differences in growth and luciferase activity between *CXCL10* virus-infected and non-infected luciferase-expressing OSA cells made it difficult to assess tumor burden by *in vivo* luminescence imaging. As a result, we used a luciferase non-expressing subcutaneous model, which does not spontaneously metastasize until late stages. A recent study reported that CXCR3A on OSA increased metastasis through CXCL10-CXCR3A signaling-mediated phosphorylation of AKT and PAK1.[45] Consistently, our scRNA-seq data also showed CXCL10-induced activation of AKT, a known effector of metastasis.[46,47] This raises a theoretical concern that CXCL10 over-secretion could drive tumor dissemination. However, activated NK cells have shown efficacy against OSA lung metastasis,[48] and silencing *CXCR3A* selectively in OSA cells may also mitigate this risk.[49–51]

In conclusion, this study presents a novel therapeutic strategy combining chemokine-driven NK cell recruitment with IL-15-based NK cell activation. Our findings demonstrated that CXCL9, -10, and -11 significantly enhanced chemotaxis of *ex vivo*-expanded CXCR3-positive NK cells into the OSA TME and that NKTR-255 further boosted their cytotoxic efficacy. These results support further investigation of engineered CXCL9, -10, or -11 secretion, particularly via oncolytic virotherapy, in combination with an IL-15 agonist to enhance the efficacy of NK cell-based therapy against solid tumors, especially OSA.

MATERIALS AND METHODS

Expansion of NK cells

NK cells were *ex vivo* expanded from human peripheral blood mononuclear cells (PBMCs) as we previously reported.[52] Briefly,

K562-mbIL21-41BBL feeder cells were irradiated and co-cultured with PBMCs at a 1:1 cell ratio in RPMI 1640 with HEPES (Thermo Fisher Scientific, Waltham, MA, USA), supplemented with 10% fetal bovine serum (FBS) (Bio-Techne, Minneapolis, MN, USA), 100 μg/mL penicillin-streptomycin (PS) (Corning, Glendale, AZ, USA), and 50 IU/mL IL-2 (Thermo Fisher Scientific). The cultures were restimulated with feeder cells at a 1:1 cell ratio on day 7. NK cells on days 14–20 of expansion were purified using the NK Cell Isolation Kit, human (Miltenyi Biotec, Auburn, CA, USA) before subsequent *in vitro* and *in vivo* experiments. IL-21, delivered through K562-mbIL21-41BBL feeder cells, and IL-2 have been reported to stimulate the JAK-STAT signaling pathway, ultimately leading to the activation of downstream transcription factors such as T-bet.[30,31] T-bet, in turn, governs the expression of chemokine receptors including CXCR3.[32] Thus, our *ex vivo* expansion protocol supports the generation of CXCR3-positive NK cells without genetic manipulation. The expression of CXCR3 on NK cells was analyzed by flow cytometry.

Cell lines

The human OSA cell lines 143B (RRID: CVCL_2270) and U2OS (RRID: CVCL_0042) were obtained from American Type Culture Collection (https://www.atcc.org). 143B was maintained in Minimum Essential Medium Eagle (Sigma-Aldrich, St. Louis, MO, USA), supplemented with 10% FBS (Bio-Techne), 100 μg/mL PS (Corning), and 0.015 mg/mL 5-bromo-2′-deoxyuridine (Sigma-Aldrich). U2OS was maintained in Dulbecco's Modified Eagle's Medium (DMEM) (Corning), supplemented with 10% FBS (Bio-Techne), 100 μg/mL PS (Corning), and 1 mM sodium pyruvate (Thermo Fisher Scientific). Cell lines were tested quarterly for mycoplasma contamination using the MycoProbe Mycoplasma Detection Kit (R&D Systems, Minneapolis, MN, USA), and cells were authenticated by short tandem repeat profiling (Genetica DNA Laboratories, Cincinnati, OH, USA).

DNA constructs

CXCL9, *-10*, and *-11* genes were synthesized (GenScript) and cloned into pcDNA3 (Thermo Fisher Scientific) via HindIII and XhoI sites. For cloning into retroviral vectors, *CXCL9*, *-10*, and *-11* genes were released from pcDNA3 using HindIII (New England Biolabs, Ipswich, MA, USA) and XhoI (New England Biolabs) and blunt-ended before ligating into pMSCVneo (Clontech, Mountain View, CA, USA) or pMSCVhygro (Clontech), digested with EcoRI (New England Biolabs) or XhoI (New England Biolabs), respectively, and blunt-ended.

Retroviral transduction

All experiments were approved by the Institutional Biosafety Committee at New York Medical College (Protocol #07-2023-2). Retroviruses were produced by transfection of human embryonic kidney 293T cells with *CXCL9*, *-10*, or *-11*-pMSCVneo or pMSCVhygro plasmids, along with GAG/POL and VSV-G plasmids. 143B and U2OS cells were transduced with viruses in the presence of 10 μg/mL polybrene (Sigma-Aldrich), and transduced tumor cells were selected by geneticin (G418 sulfate; Corning) or hygromycin B (Sigma-Aldrich), respectively. The production of CXCL9, -10, and -11 by tumor cell lines was confirmed by the enzyme-linked immunosorbent assay (ELISA) after at least 5 days of selection by geneticin or hygromycin B. For luciferase-based analysis, 143B and U2OS cells were transduced with a virus encoding luciferase as we previously reported.[22] The expression of luciferase was confirmed during the luciferase-based cytotoxicity assay after at least 5 days of selection by geneticin.

Preparation of conditioned media

CXCL9, *-10*, or *-11* virus-infected or non-infected 143B cells were seeded at 2.5×10^6 cells per well in a 6-well plate and allowed to adhere overnight. The following day, the media were replaced with 2 mL of serum-free Minimum Essential Medium Eagle (Sigma-Aldrich) and cultured for 6 h. *CXCL9*, *-10*, or *-11* virus-infected or non-infected U2OS cells were seeded at 2.5×10^6 cells per well in a 6-well plate and allowed to adhere overnight. The following day, the media were replaced with 2 mL of serum-free DMEM (Corning) and cultured for 6 h. The conditioned media were collected and centrifuged to remove tumor cells. The supernatants were used for the chemokine production assay and transwell assay.

Chemokine production assay

CXCL9, -10, and -11 levels in the conditioned media were evaluated using the ELISA Kits (Bio-Techne) according to the manufacturer's instructions. The minimum detectable doses of CXCL9, -10, and -11 range 1.37–11.31 pg/mL (mean 3.84 pg/mL), 0.41–4.46 pg/mL (mean 1.67 pg/mL), and 3.4–39.7 pg/mL (mean 13.9 pg/mL), respectively.

Transwell assay

Conditioned media (600 μL) were transferred to a 24-well plate. Transwell inserts with polycarbonate membranes (5 μm pore size; MilliporeSigma, Burlington, MA, USA) were placed in the wells containing conditioned media. NK cells (5×10^5) in 100 μL serum-free media were added to the inserts and incubated at 37°C for 2 h. Following incubation, migrated cells from the lower chamber were counted.

Luciferase-based cytotoxicity assay

The luciferase-based *in vitro* cytotoxicity assay was performed as we have previously described.[21] Briefly, *CXCL9*, *-10*, or *-11* virus-infected or non-infected luciferase-expressing 143B or U2OS cells (5×10^4/well) were seeded in a 96-well plate and incubated at 37°C for 18–19 h with NK cells at different E:T ratios. D-firefly luciferin potassium salt (GoldBio, St Louis, MO, USA) was added to the cells, and bioluminescence was measured with a luminometer (FilterMax F5 Multi-Mode Microplate Reader; Molecular Devices, San Jose, CA, USA). Target cell lysis was defined as the difference between luciferase activity of 143B or U2OS cells cultured alone and residual luciferase activity after co-culture with NK cells. To investigate the effect of NKTR-255 on NK cytotoxicity, NK cells were cultured in IL-2-free media with or without NKTR-255 (40 ng/mL, generously

provided by Nektar Therapeutics) for 2 days before incubating with luciferase-expressing 143B cells.

Tumor cell proliferation assay

CXCL10 virus-infected or non-infected, luciferase-expressing or non-expressing 143B cells (5×10^5/well) were seeded in a 6-well plate. Cells were counted and reseeded every 3 days for 15 days.

Xenograft models

All experiments were approved by the Institutional Animal Care and Use Committee at New York Medical College (Protocol #20860) and conformed to all relevant regulatory standards.

WT and chemokine (CXCL9, -10, or -11)-secreting 143B cells were injected (s.c. 2×10^6/site) in each flank of the same male NSG mouse at 5 weeks of age (bred and maintained at New York Medical College). For migration experiments, luciferase-expressing human NK cells were injected (i.p. 2.7×10^6) 5 days following tumor inoculation. Mice were injected i.p. with 150 mg/kg of D-luciferin salt (GoldBio) and imaged using the IVIS Spectrum (Caliper Life Sciences, Hopkinton, MA, USA) 1–3 h following injection with NK cells. For infiltration experiments, human NK cells were injected (i.p. 1.5×10^7) 5–6 days following tumor inoculation. To maintain NK cell longevity *in vivo*, all mice received an i.p. injection of NKTR-255 (0.3 mg/kg, generously provided by Nektar Therapeutics) on the day of NK cell infusions. Tumors were resected 5–7 days following injection with NK cells. The tissues were analyzed by flow cytometry and immunohistochemical staining (supplemental materials and methods).

For tumor progression and survival analysis, WT or *CXCL10*-transduced 143B cells were injected (s.c. 5×10^5/mouse) in the left flank of either male or female NSG mice at 4–9 weeks of age (the sex and age were similarly distributed among groups). Tumor-bearing mice were divided into 4 treatment groups: (1) PBS control, (2) NKTR-255, (3) expanded NK, and (4) expanded NK and NKTR-255. One day after tumor inoculation, expanded human NK cells (1×10^7) and NKTR-255 (0.3 mg/kg) were injected i.p., followed by NK cell injection once a week and NKTR-255 injection once every 3 weeks as we previously reported[21,22] with modification. Mice not injected with NK cells or NKTR-255 received i.p. injections of 100 μL PBS. Treatment response was evaluated through tumor progression measured with a digital caliper every 7 days. Tumor volume was obtained through the formula (length $\times$ width^2)/2. No randomization or blinding was used for grouping, treatment, or tumor measurement. The endpoint of these experiments was the number of days from tumor inoculation to death or euthanization (censoring) of animals with tumor size(s) of over 2 cm^3 or severe ulceration on tumors in accordance with our institutional guidelines. The decision to euthanize mice was made by the staff at the animal facility, who were completely blinded to the study.

For analysis of response and resistance mechanisms, WT and CXCL10-secreting 143B cells were injected (s.c. 5×10^5/site) in each flank of the same either male or female NSG mouse at 6–9 weeks of age (the sex and age were similarly distributed among groups). Tumor-bearing mice were divided into 2 treatment groups: (1) PBS control and (2) expanded NK and NKTR-255. One day after tumor inoculation, expanded human NK cells (1.5×10^7) and NKTR-255 (0.3 mg/kg) were injected i.p., followed by NK cell injection once a week as we have previously reported[21,22] with modification. Mice not injected with NK cells and NKTR-255 received i.p. injections of 100 μL PBS. Tumors were harvested 21–23 days following tumor injection. Dissociated tumors were analyzed by scRNA-seq and mass cytometry.

Preparation of single-cell suspensions

Tumors were dissociated using the Tumor Dissociation Kit, mouse (Miltenyi Biotec) according to the manufacturer's protocol. The cell suspension was passed through a 70 μm cell strainer (Corning) and used for flow cytometry or further purification steps described below.

For scRNA-seq and mass cytometry, red blood cells were removed from the cell suspension using RBC Lysis Solution (BioLegend, San Diego, CA, USA). The cell suspension was passed through a 40 μm Flowmi Cell Strainer (Bel-Art, Wayne, NJ, USA). Dead cells were removed using the Dead Cell Removal Kit (Miltenyi Biotec) according to the manufacturer's protocol.

Flow cytometry

The expression of CXCR3 on naive, fresh expanded, or cryopreserved expanded NK cells was analyzed using an antibody against CXCR3 (BD Biosciences, Franklin Lakes, NJ, USA, Cat# 560831; RRID: AB_2033944). 10,000 events per sample were acquired on the BD FACSCanto II Flow Cytometer (BD Biosciences; RRID: SCR_018056). Infiltrated NK cells after adoptive transfer were analyzed by staining dissociated tumors with an anti-human CD45 antibody (BD Biosciences, Cat# 560973; RRID: AB_398600). 100,000 events per tumor were acquired on the flow cytometer. All flow cytometry data were analyzed using the FlowJo software (Tree Star, Ashland, OR, USA; RRID: SCR_008520).

scRNA-seq

The single-cell suspension was fixed using the Evercode Cell Fixation Kit (Parse Biosciences, Seattle, WA, USA), and libraries were constructed using the Parse Biosciences platform according to the manufacturer's protocol. One biological replicate (one tumor sample) was analyzed per condition. Each biological sample was sequenced across two lanes on the Illumina NextSeq 2000 system (Illumina, San Diego, CA, USA; RRID: SCR_023614), and data from both lanes were merged prior to analysis. All cells that passed the initial sequencing quality control (QC) (as defined by the Parse Biosciences pipeline) from each merged sample (Table S1) were imported into Seurat v.5 in R.[53] Cells were filtered by three QC thresholds (percentage of mitochondrial genes <20%, nCount_RNA <25,000, nFeature_RNA >200 and <5,000). Sequencing reads were aligned to human and mouse mixed genome to distinguish species origin. A cutoff of >75% of reads/unique molecular identifiers was used to assign

mouse and human cells. Separated human and mouse cells were analyzed independently. The Seurat FindAllMarkers function was used to identify cluster-specific marker genes for cell type annotation using the rPanglaoDB R package.[54] Following data processing using standard Seurat methods, differential gene expression analysis between experimental groups was performed using the Seurat FindMarkers function. GSEA was performed on −log(p value) *sign(logFC) for each comparison using the fgsea R package[55] on gene sets (canonical pathways) from the Molecular Signatures Database.[56,57]

Mass cytometry

Single cells were stained with metal-labeled antibodies including an apoptotic marker (cPARP), human NK cell markers (CD56, CD94, CD16, CD107a, NKp30, NKG2A, NKG2D, TIGIT, CD69, and Perforin), and murine myeloid markers (CD45.1, CD11b, F4/80, Ly6G, CD44, CD206, and CD69) (Table S2) and fixed (supplemental materials and methods). All events (Table S1) in each sample were acquired on the Fluidigm CyTOF Helios Mass Cytometer (Fluidigm, South San Francisco, CA, USA; RRID: SCR_019916).

Statistics

Data are represented as mean ± standard deviation (SD). As indicated in each figure legend, the Student's t test or analysis of variance (ANOVA) with post hoc Bonferroni test was used to calculate statistically significant differences between groups. *In vivo* tumor growth was analyzed by a mixed-effects model, accounting for observational dependencies for each subject. ANOVA and mixed-effects modeling were conducted using SAS 9.4 (SAS Institute, Cary, NC, USA). For mouse survival experiments, survival probabilities were determined by the Kaplan-Meier method using GraphPad Prism (GraphPad Software, Boston, MA, USA; RRID: SCR_002798). Statistical analysis of survival between groups was performed using the log rank test. A p value of <0.05 was considered statistically significant in all experiments. Before conducting mouse experiments, sample sizes achieving 80% power to detect an effect size >2 were determined at a significance level as 0.05 using Power Analysis and Sample Size software (NCSS LLC, Kaysville, UT, USA; RRID: SCR_019099).

DATA AVAILABILITY

Data and materials are available from the corresponding author on reasonable request. The scRNA-seq data generated and/or analyzed during the current study are available in the Gene Expression Omnibus (GEO) repository, GEO: GSE297427.

ACKNOWLEDGMENTS

This work was primarily supported by a grant from the National Cancer Institute Cancer Moonshot U54 CA23256 (M.S.C. and D.A.L.). Additional support was from the St. Baldrick's Foundation (M.S.C. and D.A.L.), Pediatric Cancer Research Foundation (M.S. C.), and Children's Cancer Fund (M.S.C.). We would like to acknowledge the staff at the Cellular Tissue Engineering Laboratory, the Children and Adolescent Cancer & Blood Disease Center, Westchester Medical Center; the staff at the animal facility at the Department of Comparative Medicine, New York Medical College; Ravi Sachidanandam, PhD at the Department of Pathology, Microbiology, and Immunology, New York Medical College, for scRNA-seq data analysis; Justin Lyberger at the Division of Hematology, The Ohio State University, for mass cytometry data acquisition; Yanling Liao, PhD, Jian Pan, Morgan Anderson-Crannage, and Mishel Ramirez at the Department of Pediatrics, New York Medical College, for technical support; and Virginia Davenport, RN, and Erin A. Morris, RN, BSN, at the Department of Pediatrics, New York Medical College, for the preparation and submission of this manuscript. The graphical abstract was created in BioRender.

This work in part was presented at the American Association for Cancer Research Annual Meeting 2025, April 25–30, 2025, in Chicago, Illinois, USA.

AUTHOR CONTRIBUTIONS

S.E., W.L., and M.S.C. contributed to the study conception and design. Material preparation, data collection, and analysis were performed by S.E., W.L., H.Z., H.M.H., C.X., G.K.B., K.L.T., and J.A. The first draft of the manuscript was written by S.E. and W.L. M.S.C. supervised the study. G.K.B., M.M., and D.A.L. provided key reagents and/or contributed intellectually. All authors reviewed and approved the final manuscript.

DECLARATION OF INTERESTS

M.S.C. has served as a consultant for Jazz Pharmaceuticals, Omeros Pharmaceuticals, and AbbVie; served on the Speakers Bureau for Jazz Pharmaceuticals and Amgen; and received research funding from Merck, Miltenyi Biotec, Servier, Omeros, Jazz, and Janssen. D.A.L. reports personal fees and others from Kiadis Pharma, CytoSen Therapeutics, Courier Therapeutics, and Caribou Biosciences outside the submitted work. In addition, D.A.L. has a patent broadly related to NK cell therapy of cancer with royalties paid to Kiadis Pharma. M.M. is an employee of Nektar Therapeutics.

SUPPLEMENTAL INFORMATION

Supplemental information can be found online at https://doi.org/10.1016/j.omton.2025.201051.

REFERENCES

1. Bishop, M.W., Janeway, K.A., and Gorlick, R. (2016). Future directions in the treatment of osteosarcoma. Curr. Opin. Pediatr. *28*, 26–33. https://doi.org/10.1097/MOP.0000000000000298.

2. Robbins, G.M., Vue, Y.Y., Rahrmann, E.P., and Moriarity, B.S. (2024). Osteosarcoma: A comprehensive review of model systems and experimental therapies. Med. Res. Arch. *12*, 6000. https://doi.org/10.18103/mra.v12i11.6000.

3. Gelderblom, H., Jinks, R.C., Sydes, M., Bramwell, V.H.C., van Glabbeke, M., Grimer, R.J., Hogendoorn, P.C.W., McTiernan, A., Lewis, I.J., Nooij, M.A., et al. (2011). Survival after recurrent osteosarcoma: data from 3 European Osteosarcoma Intergroup (EOI) randomized controlled trials. Eur. J. Cancer *47*, 895–902. https://doi.org/10.1016/j.ejca.2010.11.036.

4. Spraker-Perlman, H.L., Barkauskas, D.A., Krailo, M.D., Meyers, P.A., Schwartz, C.L., Doski, J., Gorlick, R., Janeway, K.A., and Isakoff, M.S. (2019). Factors influencing survival after recurrence in osteosarcoma: A report from the Children's Oncology Group. Pediatr. Blood Cancer *66*, e27444. https://doi.org/10.1002/pbc.27444.

5. Jarrell, D.K., Drake, S., and Brown, M.A. (2020). Advancing Therapies for Cancer—From Mustard Gas to CAR T. Sci *2*, 90. https://doi.org/10.3390/sci2040090.

6. Grobner, S.N., Worst, B.C., Weischenfeldt, J., Buchhalter, I., Kleinheinz, K., Rudneva, V.A., Johann, P.D., Balasubramanian, G.P., Segura-Wang, M., Brabetz, S., et al. (2018). The landscape of genomic alterations across childhood cancers. Nature *555*, 321–327. https://doi.org/10.1038/nature25480.

7. Kaczanowska, S., Murty, T., Alimadadi, A., Contreras, C.F., Duault, C., Subrahmanyam, P.B., Reynolds, W., Gutierrez, N.A., Baskar, R., Wu, C.J., et al. (2024). Immune determinants of CAR-T cell expansion in solid tumor patients receiving GD2 CAR-T cell therapy. Cancer Cell *42*, 35–51.e8. https://doi.org/10.1016/j.ccell.2023.11.011.

8. Vivier, E., Ugolini, S., Blaise, D., Chabannon, C., and Brossay, L. (2012). Targeting natural killer cells and natural killer T cells in cancer. Nat. Rev. Immunol. *12*, 239–252. https://doi.org/10.1038/nri3174.

9. Luksch, R., Perotti, D., Cefalo, G., Gambacorti Passerini, C., Massimino, M., Spreafico, F., Casanova, M., Ferrari, A., Terenziani, M., Polastri, D., et al. (2003). Immunomodulation in a treatment program including pre- and post-operative

interleukin-2 and chemotherapy for childhood osteosarcoma. Tumori *89*, 263–268. https://doi.org/10.1177/030089160308900306.

10. Yang, X., Zhang, W., and Xu, P. (2019). NK cell and macrophages confer prognosis and reflect immune status in osteosarcoma. J. Cell. Biochem. *120*, 8792–8797. https://doi.org/10.1002/jcb.28167.
11. Markiewicz, K., Zeman, K., Kozar, A., Gołębiowska-Wawrzyniak, M., and Woźniak, W. (2014). [Evaluation of selected parameters of specific immunity in children with osteosarcoma at various stages of antitumour treatment]. Dev. Period Med. *18*, 155–168.
12. Tong, L., Jiménez-Cortegana, C., Tay, A.H.M., Wickström, S., Galluzzi, L., and Lundqvist, A. (2022). NK cells and solid tumors: therapeutic potential and persisting obstacles. Mol. Cancer *21*, 206. https://doi.org/10.1186/s12943-022-01672-z.
13. Portillo, A.L., Monteiro, J.K., Rojas, E.A., Ritchie, T.M., Gillgrass, A., and Ashkar, A. A. (2023). Charting a killer course to the solid tumor: strategies to recruit and activate NK cells in the tumor microenvironment. Front. Immunol. *14*, 1286750. https://doi.org/10.3389/fimmu.2023.1286750.
14. Denman, C.J., Senyukov, V.V., Somanchi, S.S., Phatarpekar, P.V., Kopp, L.M., Johnson, J.L., Singh, H., Hurton, L., Maiti, S.N., Huls, M.H., et al. (2012). Membrane-bound IL-21 promotes sustained *ex vivo* proliferation of human natural killer cells. PLoS One *7*, e30264. https://doi.org/10.1371/journal.pone.0030264.
15. Chu, Y., Hochberg, J., Yahr, A., Ayello, J., van de Ven, C., Barth, M., Czuczman, M., and Cairo, M.S. (2015). Targeting CD20+ Aggressive B-cell Non-Hodgkin Lymphoma by Anti-CD20 CAR mRNA-Modified Expanded Natural Killer Cells In Vitro and in NSG Mice. Cancer Immunol. Res. *3*, 333–344. https://doi.org/10.1158/2326-6066.CIR-14-0114.
16. Conlon, K.C., Lugli, E., Welles, H.C., Rosenberg, S.A., Fojo, A.T., Morris, J.C., Fleisher, T.A., Dubois, S.P., Perera, L.P., Stewart, D.M., et al. (2015). Redistribution, hyperproliferation, activation of natural killer cells and CD8 T cells, and cytokine production during first-in-human clinical trial of recombinant human interleukin-15 in patients with cancer. J. Clin. Oncol. *33*, 74–82. https://doi.org/10.1200/JCO.2014.57.3329.
17. Suck, G., Oei, V.Y.S., Linn, Y.C., Ho, S.H., Chu, S., Choong, A., Niam, M., and Koh, M.B.C. (2011). Interleukin-15 supports generation of highly potent clinical-grade natural killer cells in long-term cultures for targeting hematological malignancies. Exp. Hematol. *39*, 904–914. https://doi.org/10.1016/j.exphem.2011.06.003.
18. Miyazaki, T., Maiti, M., Hennessy, M., Chang, T., Kuo, P., Addepalli, M., Obalapur, P., Sheibani, S., Wilczek, J., Pena, R., et al. (2021). NKTR-255, a novel polymer-conjugated rhIL-15 with potent antitumor efficacy. J. Immunother. Cancer *9*, e002024. https://doi.org/10.1136/jitc-2020-002024.
19. Shah, N., Perales, M.A., Turtle, C.J., Cairo, M.S., Cowan, A.J., Saeed, H., Budde, L.E., Tan, A., Lee, Z., Kai, K., et al. (2021). Phase I study protocol: NKTR-255 as monotherapy or combined with daratumumab or rituximab in hematologic malignancies. Future Oncol. *17*, 3549–3560. https://doi.org/10.2217/fon-2021-0576.
20. Shah, N., Tan, A., Budde, L.E., Hoffmeister, C.C., Cowan, A.J., Perales, M.A., Choi, T., Saeed, H., Chavez, J.C., Ye, J.C., et al. (2021). Safety, tolerability, PK/PD and preliminary efficacy of NKTR-255, a novel IL-15 receptor agonist. In Patients with Relapsed/refractory Hematologic Malignancies (Blood).
21. Luo, W., Gardenswartz, A., Hoang, H., Chu, Y., Tian, M., Liao, Y., Ayello, J., Rosenblum, J.M., Mo, X., Marcondes, A.M., et al. (2024). Combinatorial immunotherapy of anti-MCAM CAR-modified expanded natural killer cells and NKTR-255 against neuroblastoma. Mol. Ther. Oncol. *32*, 200894. https://doi.org/10.1016/j.omton.2024.200894.
22. Luo, W., Hoang, H., Zhu, H., Miller, K., Mo, X., Eguchi, S., Tian, M., Liao, Y., Ayello, J., Rosenblum, J.M., et al. (2024). Circumventing resistance within the Ewing sarcoma microenvironment by combinatorial innate immunotherapy. J. Immunother. Cancer *12*, e009726. https://doi.org/10.1136/jitc-2024-009726.
23. Kohli, K., Pillarisetty, V.G., and Kim, T.S. (2022). Key chemokines direct migration of immune cells in solid tumors. Cancer Gene Ther. *29*, 10–21. https://doi.org/10.1038/s41417-021-00303-x.
24. Lasagni, L., Francalanci, M., Annunziato, F., Lazzeri, E., Giannini, S., Cosmi, L., Sagrinati, C., Mazzinghi, B., Orlando, C., Maggi, E., et al. (2003). An alternatively spliced variant of CXCR3 mediates the inhibition of endothelial cell growth induced by IP-10, Mig, and I-TAC, and acts as functional receptor for platelet factor 4. J. Exp. Med. *197*, 1537–1549. https://doi.org/10.1084/jem.20021897.
25. Liu, E., Marin, D., Banerjee, P., Macapinlac, H.A., Thompson, P., Basar, R., Nassif Kerbauy, L., Overman, B., Thall, P., Kaplan, M., et al. (2020). Use of CAR-Transduced Natural Killer Cells in CD19-Positive Lymphoid Tumors. N. Engl. J. Med. *382*, 545–553. https://doi.org/10.1056/NEJMoa1910607.
26. Marin, D., Li, Y., Basar, R., Rafei, H., Daher, M., Dou, J., Mohanty, V., Dede, M., Nieto, Y., Uprety, N., et al. (2024). Safety, efficacy and determinants of response of allogeneic CD19-specific CAR-NK cells in CD19(+) B cell tumors: a phase 1/2 trial. Nat. Med. *30*, 772–784. https://doi.org/10.1038/s41591-023-02785-8.
27. Ciurea, S.O., Kongtim, P., Srour, S., Chen, J., Soebbing, D., Shpall, E., Rezvani, K., Nakkula, R., Thakkar, A., Troy, E.C., et al. (2024). Results of a phase I trial with Haploidentical mbIL-21 ex vivo expanded NK cells for patients with multiply relapsed and refractory AML. Am. J. Hematol. *99*, 890–899. https://doi.org/10.1002/ajh.27281.
28. Ciurea, S.O., Kongtim, P., Soebbing, D., Trikha, P., Behbehani, G., Rondon, G., Olson, A., Bashir, Q., Gulbis, A.M., Indreshpal, K., et al. (2022). Decrease post-transplant relapse using donor-derived expanded NK-cells. Leukemia *36*, 155–164. https://doi.org/10.1038/s41375-021-01349-4.
29. Tarannum, M., Romee, R., and Shapiro, R.M. (2022). Innovative Strategies to Improve the Clinical Application of NK Cell-Based Immunotherapy. Front. Immunol. *13*, 859177. https://doi.org/10.3389/fimmu.2022.859177.
30. Huang, C., and Bi, J. (2021). Expression Regulation and Function of T-Bet in NK Cells. Front. Immunol. *12*, 761920. https://doi.org/10.3389/fimmu.2021.761920.
31. Gotthardt, D., Trifinopoulos, J., Sexl, V., and Putz, E.M. (2019). JAK/STAT Cytokine Signaling at the Crossroad of NK Cell Development and Maturation. Front. Immunol. *10*, 2590. https://doi.org/10.3389/fimmu.2019.02590.
32. Matsuda, J.L., George, T.C., Hagman, J., and Gapin, L. (2007). Temporal dissection of T-bet functions. J. Immunol. *178*, 3457–3465. https://doi.org/10.4049/jimmunol.178.6.3457.
33. Ali, A., Canaday, L.M., Feldman, H.A., Cevik, H., Moran, M.T., Rajaram, S., Lakes, N., Tuazon, J.A., Seelamneni, H., Krishnamurthy, D., et al. (2021). Natural killer cell immunosuppressive function requires CXCR3-dependent redistribution within lymphoid tissues. J. Clin. Investig. *131*, e146686. https://doi.org/10.1172/JCI146686.
34. Wang, X., Zhang, Y., Wang, S., Ni, H., Zhao, P., Chen, G., Xu, B., and Yuan, L. (2022). The role of CXCR3 and its ligands in cancer. Front. Oncol. *12*, 1022688. https://doi.org/10.3389/fonc.2022.1022688.
35. Talbot, L.J., Chabot, A., Ross, A.B., Beckett, A., Nguyen, P., Fleming, A., Chockley, P. J., Shepphard, H., Wang, J., Gottschalk, S., and DeRenzo, C. (2024). Redirecting B7-H3.CAR T Cells to Chemokines Expressed in Osteosarcoma Enhances Homing and Antitumor Activity in Preclinical Models. Clin. Cancer Res. *30*, 4434–4449. https://doi.org/10.1158/1078-0432.CCR-23-3298.
36. Zhou, Y., Yang, D., Yang, Q., Lv, X., Huang, W., Zhou, Z., Wang, Y., Zhang, Z., Yuan, T., Ding, X., et al. (2020). Single-cell RNA landscape of intratumoral heterogeneity and immunosuppressive microenvironment in advanced osteosarcoma. Nat. Commun. *11*, 6322. https://doi.org/10.1038/s41467-020-20059-6.
37. Colvin, R.A., Campanella, G.S.V., Sun, J., and Luster, A.D. (2004). Intracellular domains of CXCR3 that mediate CXCL9, CXCL10, and CXCL11 function. J. Biol. Chem. *279*, 30219–30227. https://doi.org/10.1074/jbc.M403595200.
38. Souza-Fonseca-Guimaraes, F., Rossi, G.R., Dagley, L.F., Foroutan, M., McCulloch, T.R., Yousef, J., Park, H.Y., Gunter, J.H., Beavis, P.A., Lin, C.Y., et al. (2022). TGFbeta and CIS Inhibition Overcomes NK-cell Suppression to Restore Antitumor Immunity. Cancer Immunol. Res. *10*, 1047–1054. https://doi.org/10.1158/2326-6066.CIR-21-1052.
39. Foltz, J.A., Moseman, J.E., Thakkar, A., Chakravarti, N., and Lee, D.A. (2018). TGFbeta Imprinting During Activation Promotes Natural Killer Cell Cytokine Hypersecretion. Cancers (Basel) *10*, 423. https://doi.org/10.3390/cancers10110423.
40. Chen, J., Liao, S., Xiao, Z., Pan, Q., Wang, X., Shen, K., Wang, S., Yang, L., Guo, F., Liu, H.F., and Pan, Q. (2022). The development and improvement of immunodeficient mice and humanized immune system mouse models. Front. Immunol. *13*, 1007579. https://doi.org/10.3389/fimmu.2022.1007579.
41. Perna, S.K., De Angelis, B., Pagliara, D., Hasan, S.T., Zhang, L., Mahendravada, A., Heslop, H.E., Brenner, M.K., Rooney, C.M., Dotti, G., and Savoldo, B. (2013).

Interleukin 15 provides relief to CTLs from regulatory T cell-mediated inhibition: implications for adoptive T cell-based therapies for lymphoma. Clin. Cancer Res. *19*, 106–117. https://doi.org/10.1158/1078-0432.CCR-12-2143.

42. Gillgrass, A., Gill, N., Babian, A., and Ashkar, A.A. (2014). The absence or overexpression of IL-15 drastically alters breast cancer metastasis via effects on NK cells, CD4 T cells, and macrophages. J. Immunol. *193*, 6184–6191. https://doi.org/10.4049/jimmunol.1303175.

43. Karadimas, T., Huynh, T.H., Chose, C., Zervoudakis, G., Clampitt, B., Lapp, S., Joyce, D., Letson, G.D., Metts, J., Binitie, O., et al. (2024). Oncolytic Viral Therapy in Osteosarcoma. Viruses *16*, 1139. https://doi.org/10.3390/v16071139.

44. Chu, Y., Tian, M., Saini, U., Ayala-Cuesta, J., Klose, K., Mendelowitz, A.S., Foley, K., Ozkaynak, M.F., Luo, W., Cripe, T.P., et al. (2025). Combinatorial immunotherapy with anti-ROR1 CAR NK cells and an IL-21 secreting oncolytic virus against neuroblastoma. Mol. Ther. Oncol. *33*, 200927. https://doi.org/10.1016/j.omton.2024.200927.

45. Gyau, B.B., Wang, J., Chen, X., Clement, M.A., Man, Z.D., Major, A.M., Weiser, M. C., Xu, J., Hicks, J., and Man, T.K. (2025). The metastatic role of the CXCL10-CXCR3 axis and its therapeutic potential in osteosarcoma. J. Bone Oncol. *52*, 100690. https://doi.org/10.1016/j.jbo.2025.100690.

46. Tian, H., Lian, R., Li, Y., Liu, C., Liang, S., Li, W., Tao, T., Wu, X., Ye, Y., Yang, X., et al. (2020). AKT-induced lncRNA VAL promotes EMT-independent metastasis through diminishing Trim16-dependent Vimentin degradation. Nat. Commun. *11*, 5127. https://doi.org/10.1038/s41467-020-18929-0.

47. Zhang, Q., Tang, X., Zhou, Y., Chen, X., Peng, K., Jiang, R., Liu, Z., Song, X., and Xia, H. (2023). LINC01060 knockdown inhibits osteosarcoma cell malignant behaviors in vitro and tumor growth and metastasis in vivo through the PI3K/Akt signaling. Cancer Biol. Ther. *24*, 2198904. https://doi.org/10.1080/15384047.2023.2198904.

48. Guma, S.R., Lee, D.A., Ling, Y., Gordon, N., and Kleinerman, E.S. (2014). Aerosol interleukin-2 induces natural killer cell proliferation in the lung and combination therapy improves the survival of mice with osteosarcoma lung metastasis. Pediatr. Blood Cancer *61*, 1362–1368. https://doi.org/10.1002/pbc.25019.

49. Anesti, A.M., Simpson, G.R., Price, T., Pandha, H.S., and Coffin, R.S. (2010). Expression of RNA interference triggers from an oncolytic herpes simplex virus results in specific silencing in tumour cells in vitro and tumours in vivo. BMC Cancer *10*, 486. https://doi.org/10.1186/1471-2407-10-486.

50. Yan, Y., Liu, L., Xiong, H., Miller, J.B., Zhou, K., Kos, P., Huffman, K.E., Elkassih, S., Norman, J.W., Carstens, R., et al. (2016). Functional polyesters enable selective siRNA delivery to lung cancer over matched normal cells. Proc. Natl. Acad. Sci. USA *113*, E5702–E5710. https://doi.org/10.1073/pnas.1606886113.

51. Hu, Y., Zhang, H., Guo, Z., Zhou, J., Zhang, W., Gong, M., and Wu, J. (2023). CKM and TERT dual promoters drive CRISPR-dCas9 to specifically inhibit the malignant behavior of osteosarcoma cells. Cell. Mol. Biol. Lett. *28*, 52. https://doi.org/10.1186/s11658-023-00464-7.

52. Chu, Y., Flower, A., and Cairo, M.S. (2016). Modification of Expanded NK Cells with Chimeric Antigen Receptor mRNA for Adoptive Cellular Therapy. Methods Mol. Biol. *1441*, 215–230. https://doi.org/10.1007/978-1-4939-3684-7_18.

53. Hao, Y., Stuart, T., Kowalski, M.H., Choudhary, S., Hoffman, P., Hartman, A., Srivastava, A., Molla, G., Madad, S., Fernandez-Granda, C., and Satija, R. (2024). Dictionary learning for integrative, multimodal and scalable single-cell analysis. Nat. Biotechnol. *42*, 293–304. https://doi.org/10.1038/s41587-023-01767-y.

54. Franzen, O., Gan, L.M., and Bjorkegren, J.L.M. (2019). PanglaoDB: a web server for exploration of mouse and human single-cell RNA sequencing data. Database *2019*, baz046. https://doi.org/10.1093/database/baz046.

55. Korotkevich, G., Sukhov, V., and Sergushichev, A. (2019). Fast gene set enrichment analysis. Preprint at: bioRxiv, 060012. https://doi.org/10.1101/060012

56. Liberzon, A., Subramanian, A., Pinchback, R., Thorvaldsdóttir, H., Tamayo, P., and Mesirov, J.P. (2011). Molecular signatures database (MSigDB) 3.0. Bioinformatics *27*, 1739–1740. https://doi.org/10.1093/bioinformatics/btr260.

57. Castanza, A.S., Recla, J.M., Eby, D., Thorvaldsdóttir, H., Bult, C.J., and Mesirov, J.P. (2023). Extending support for mouse data in the Molecular Signatures Database (MSigDB). Nat. Methods *20*, 1619–1620. https://doi.org/10.1038/s41592-023-02014-7.

OMTON, Volume *33*

Supplemental information

CXCL10-induced chemotaxis of *ex vivo*-expanded natural killer cells combined with NKTR-255 enhances anti-tumor efficacy in osteosarcoma

Shiori Eguchi, Wen Luo, Hongwen Zhu, Hai M. Hoang, Changxin Xu, Gregory K. Behbehani, Kazi L. Tasneem, Janet Ayello, Mario Marcondes, Dean A. Lee, and Mitchell S. Cairo

SUPPLEMENTAL MATERIALS AND METHODS

Immunohistochemical staining

The collected tissues were embedded in Tissue-Tec OCT Compound and snap-frozen on dry ice. For each specimen, 7 μm serial sections were cut and stored at -80 °C. Sections were fixed in freshly diluted 4% paraformaldehyde (Electron Microscopy Sciences, Hatfield, PA, USA) and blocked with M.O.M. blocking reagent with 0.1% Triton (Sigma, St. Louis, MO, USA). The slides were then incubated with an anti-human CD45 antibody (1:100 dilution; Thermo Fisher Scientific, Cat# 14-9457-82; RRID: AB_11063696) and an Alexa Fluor 488-conjugated secondary antibody (1:1000 dilution; Thermo Fisher Scientific, Cat# A-11001; RRID: AB_2534069), and mounted in a Vectashield mounting medium containing DAPI (Vector Laboratories, Newark, CA, USA). Images were acquired using the EVOS M5000 imaging system (Thermo Fisher Scientific; RRID: SCR_023650).

Mass cytometry

Antibody cocktails were prepared at The Ohio State University, frozen in aliquots at -80 °C, and shipped to New York Medical College on dry ice, where they were stored at -80 °C until tumors were harvested from the experimental mice and dissociated into single-cell suspensions. Cells (1-2 $\times$ 10^6) were washed once with cell staining medium (CSM) (PBS, 0.5% bovine serum albumin) and blocked with 5 μL of Fc block (BD Biosciences, Cat# 564219; RRID: AB_2728082) diluted in 50 μL of CSM at room temperature for 10 minutes. 45 μL of the surface antibody cocktail was then added and cells were incubated at 4 °C for 1 h with periodical mixing. Cells were washed twice with CSM and fixed with 1.5% paraformaldehyde (Millipore Sigma, Burlington, MA, USA) at room temperature for 15 minutes. Cells were washed once with CSM, vortexed for 1.5 minutes, immediately resuspended with 1 mL of ice-cold methanol (Sigma-Aldrich), and incubated at -20 °C for 15 minutes. Cells were collected by centrifugation, vortexed, washed three times with CSM, and stained with 50 μL of the intracellular antibody cocktail diluted in 50 μL of CSM at 4 °C for 1 h with periodical mixing. Cells were washed once with CSM, fixed with proteomic stabilizer PROT1 (Smart Tube, Inc., Las Vegas, NV, USA) at room temperature for 10 minutes, frozen in aliquots at -80 °C, and shipped back to The Ohio State University on dry ice, where they were stored at -80 °C until they were analyzed on a mass cytometer. Samples were thawed, intercalated with Ir (191/193) intercalator (Standard Biotools, South San Francisco, CA, USA), washed into pure water with normalization beads (Standard Biotools), and then run on the Fluidigm CyTOF Helios Mass Cytometer (Fluidigm, South San Francisco, CA, USA; RRID: SCR_019916) at approximately 150-300 cell events per second. All events (Table S1) were collected from each sample. Mean ion counts for each cell events were normalized using an automated script[1] and the resulting normalized FCS files were then uploaded for all subsequent analysis on the Cytobank platform. Singlet cells were gated on plots of event length vs. Ir and offset vs. residual as previously described.[2]

Table S1. Number of events analyzed by single-cell RNA sequencing and mass cytometry

Tumor	Treatment	Analyzed events
Single-cell RNA sequencing		
WT[a]	PBS[b]	739
	NK[c] + NKTR-255	787
CXCL10	PBS	408
	NK + NKTR-255	571
Mass cytometry		
WT	PBS	80,098
		184,998
		113,219
	NK + NKTR-255	75,172
		20,411
		174,033
CXCL10	PBS	46,490
		24,899
		73,996
	NK + NKTR-255	22,313
		44,351
		36,845

[a]WT, wild-type; [b]PBS, phosphate buffer solution; [c]NK, natural killer

Table S2. List of antibodies used in mass cytometry alongside their clone information, vendor, metal label, and concentration used

Antibody	Clone	Vendor	Metal label	Concentration (μg/mL)
hCD45	HI30	BioLegend	89-Y	2
GAPDH	6c5	Invitrogen	112-Sn	2
mCD45.1	30-F11	BioLegend	115-In	1
cPARP	F21-A52	BD Biosciences	140-Ce	2
Ly6G	1A8	BioLegend	141-Pr	1
mCD11b	M1/70	BioLegend	143-Nd	1
hCD94	HP-3D9	BD Biosciences	144-Nd	2
hCD56	NCAM16.2	BD Biosciences	147-Sm	1
TIGIT	A15153G	BioLegend	150-Nd	3
HLA-DR	L243	BioLegend	153-Eu	2
hCD69	FN50	BioLegend	154-Sm	2
mCD69	H1.2F3	BioLegend	155-Gd	2
Ki-67	SolA15	Invitrogen	158-Gd	4
hNKp30	P30-15	BioLegend	159-Tb	1
hCD16	3G8	BioLegend	161-Dy	2
mCD44	IM7	BioLegend	164-Dy	1
pRb	J112-906	BD Biosciences	165-Ho	1.5
NKG2D	1D11	BioLegend	166-Er	4
mCD206	C068C2	BioLegend	167-Er	2
mF4/80	BM8	BioLegend	168-Er	2
NKG2A	S19004C	BioLegend	169-Tm	2
hCD107a	H4A3	BioLegend	170-Er	2
hPerforin	dG9	BioLegend	175-Lu	4

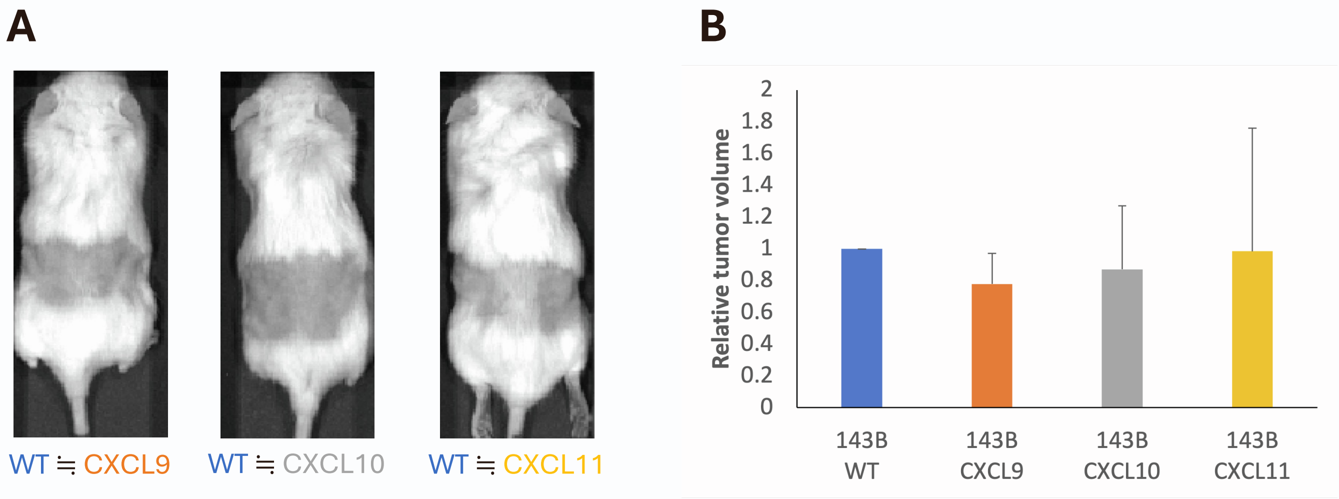

Figure S1. Similar sizes of WT and chemokine-secreting 143B tumors

NSG mice were inoculated s.c. with 2×10^6 WT and chemokine (either CXCL9, -10, or -11)-secreting 143B cells in each flank of the same mouse ($n = 3$ mice/group). Mice were imaged and tumor sizes were measured by a digital caliper 5 days following tumor inoculation.

(A) Example of tumor burden of a mouse inoculated with WT (left flank) and chemokine (either CXCL9, -10, or -11)-secreting 143B cells (right flank). One of the three representative mice/group is shown. (B) Volume of chemokine (either CXCL9, -10, or -11)-secreting tumors relative to WT tumors. Data are represented as mean ± SD. The comparison of WT and chemokine (CXCL9, -10, or -11)-secreting tumor volume was not statistically significant (Student's t test). WT, wild-type; NSG, NOD/SCID/gamma$^{-/-}$; s.c., subcutaneously; SD, standard deviation.

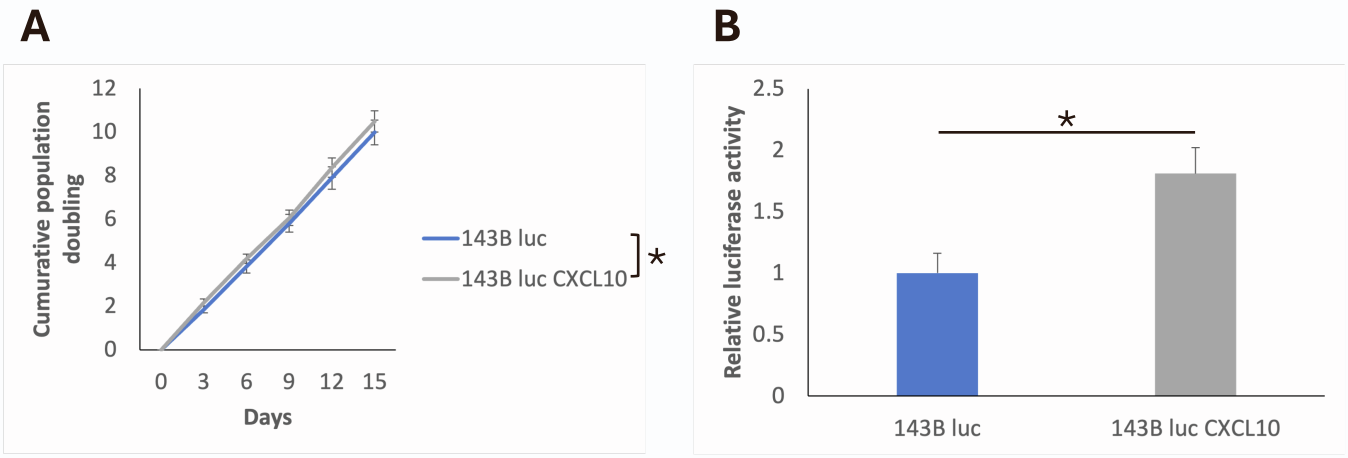

Figure S2. Significant differences in growth and luciferase activity between *CXCL10* virus-infected and non-infected 143B luc cells

(A) Growth curve of *CXCL10* virus-infected and non-infected 143B luc cells. Data are represented as mean ± SD of 3 independent biological replicates. *$p < 0.05$ (Student's t test). *CXCL10* virus-infected 143B luc cells grew significantly faster than non-infected 143B luc cells. (B) Luciferase activity of *CXCL10* virus-infected and non-infected 143B luc cells. Data are represented as mean ± SD of triplicates in a representative experiment. Experiments were repeated 3 times with similar results. *$p < 0.05$ (Student's t test). *CXCL10* virus-infected 143B luc cells had significantly higher luciferase activity than non-infected 143B luc cells. luc, luciferase-expressing; SD, standard deviation.

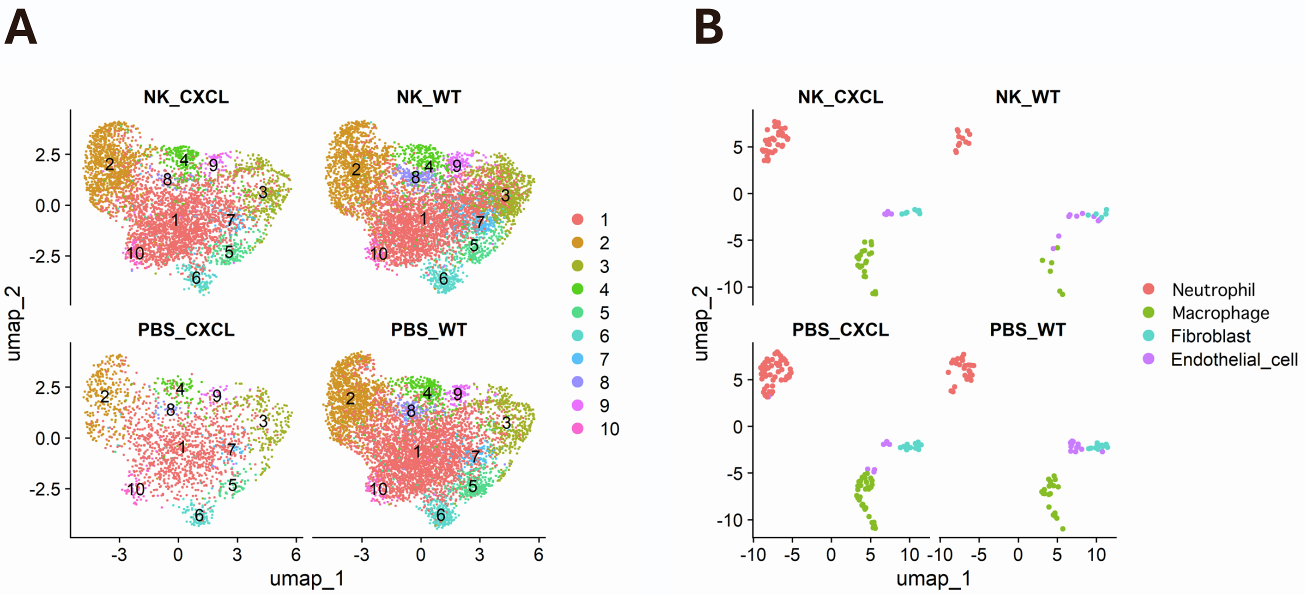

Figure S3. Clustering of all human and mouse cells
(A) UMAP displaying initial clustering of all human cells. (B) UMAP displaying initial clustering of all mouse cells. NK_CXCL, NK+NKTR-255-treated CXCL10-positive OSA; NK_WT, NK+NKTR-255-treated WT OSA; PBS_CXCL, PBS-treated CXCL10-positive OSA; PBS_WT, PBS-treated WT OSA; NK, natural killer; OSA, osteosarcoma; WT, wild-type; PBS, phosphate buffer solution; UMAP, uniform manifold approximation and projection.

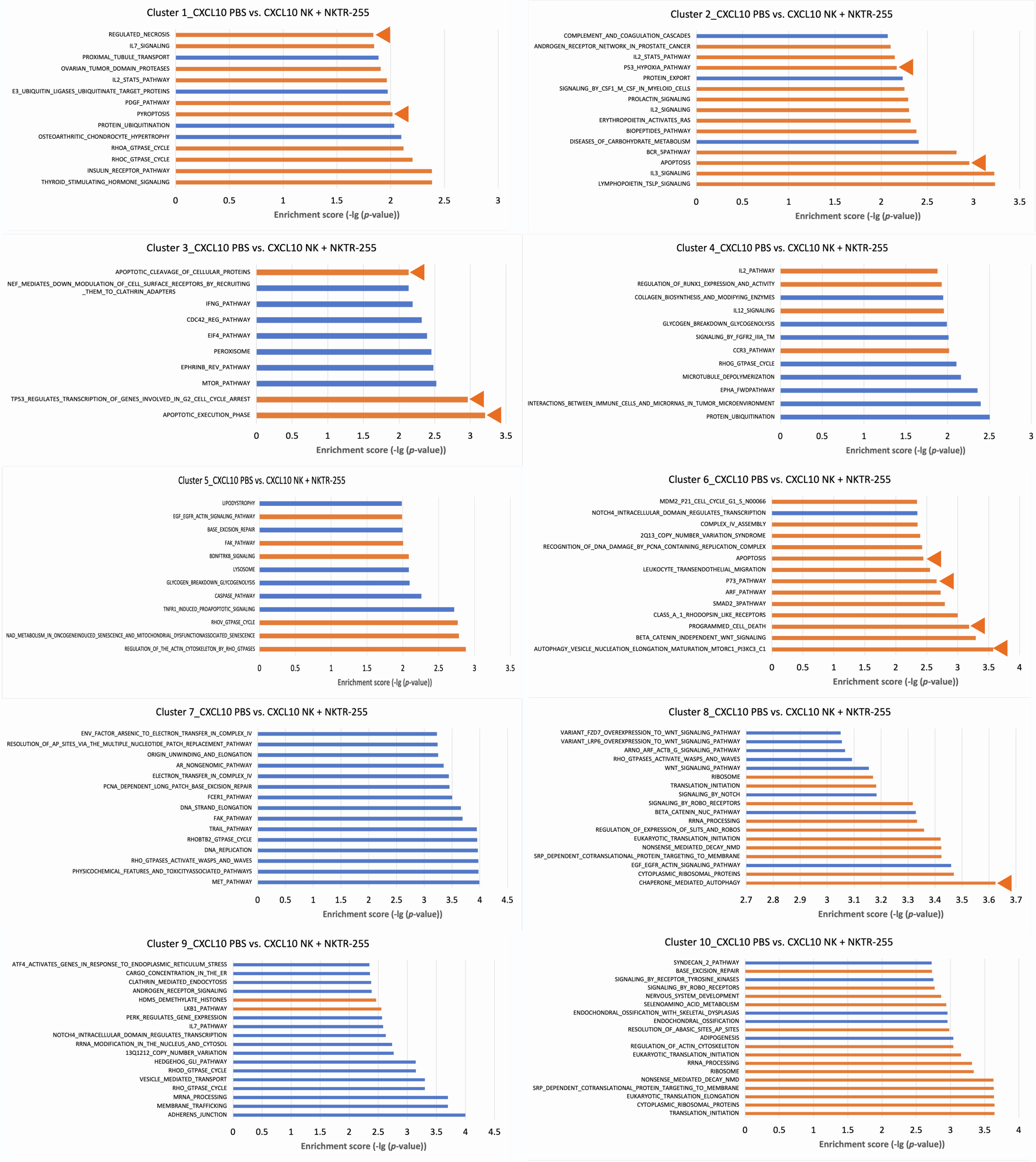

Figure S4. GSEA bar charts showing enrichment scores for the hallmark pathways that were different between CXCL10-positive tumors with and without treatment with NK cells and NKTR-255

Pathways upregulated in the CXCL10-positive tumor treated with NK cells and NKTR-255 were shown in orange. Pathways downregulated in the CXCL10-positive tumor treated with NK cells and NKTR-255 were shown in blue. Pathways indicated by arrowhead (◀) were related to cell death. GSEA, gene set enrichment analysis; NK, natural killer; PBS, phosphate buffer solution.

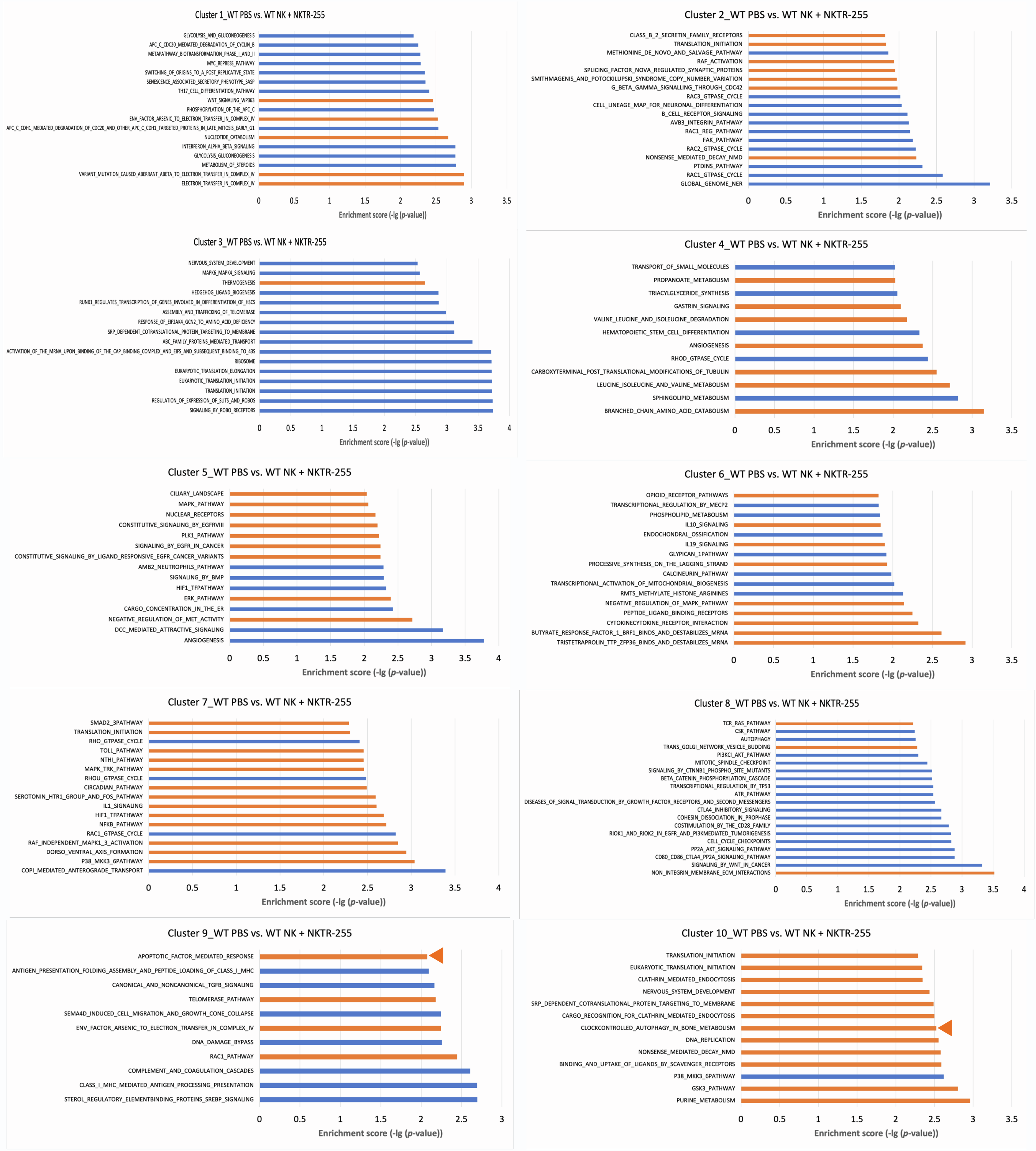

Figure S5. GSEA bar charts showing enrichment scores for the hallmark pathways that were different between WT tumors with and without treatment with NK cells and NKTR-255
Pathways upregulated in the WT tumor treated with NK cells and NKTR-255 were shown in orange. Pathways downregulated in the WT tumor treated with NK cells and NKTR-255 were shown in blue. Pathways indicated by arrowhead (◀) were related to cell death. GSEA, gene set enrichment analysis; WT, wild-type; NK, natural killer; PBS, phosphate buffer solution.

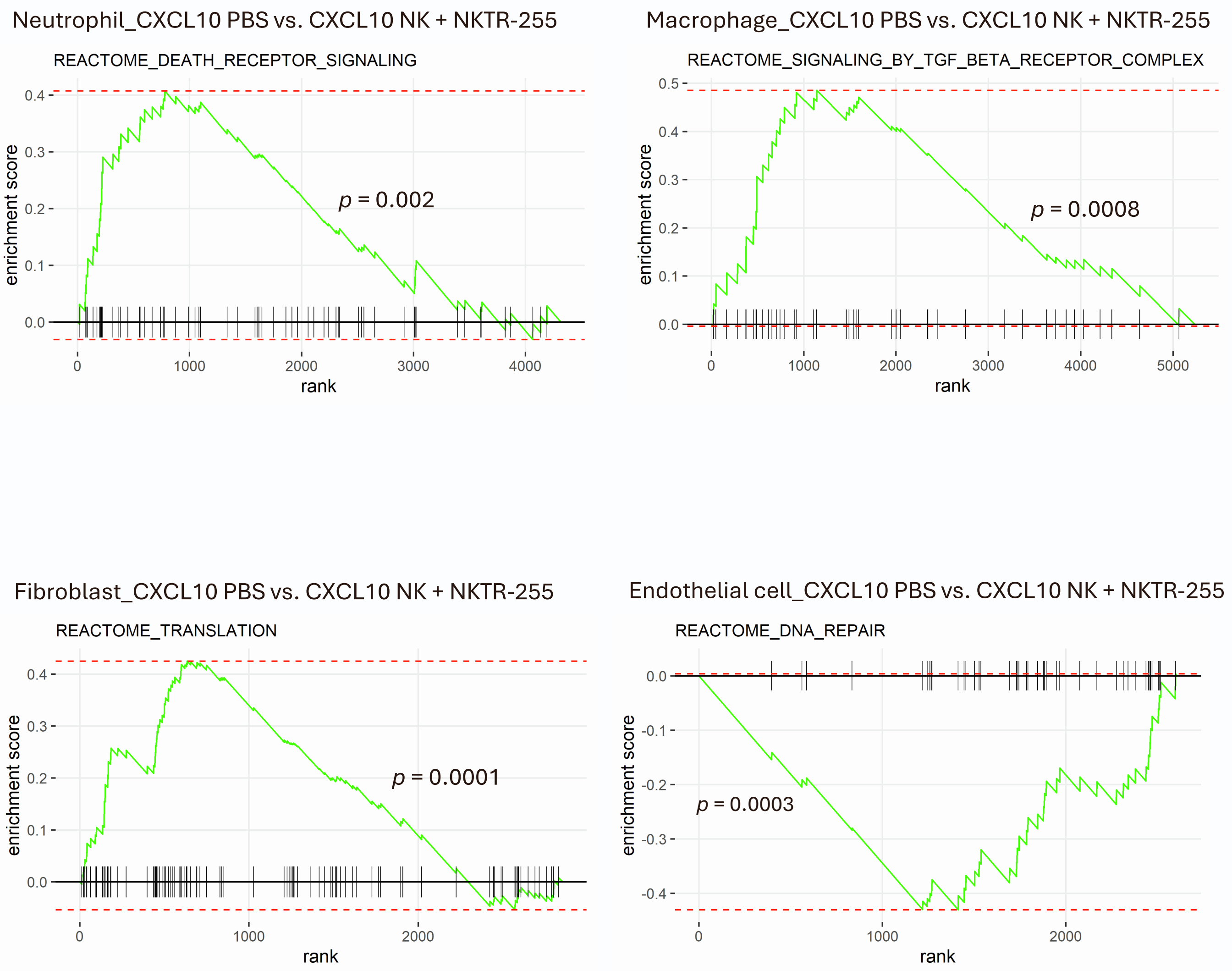

Figure S6. GSEA-enrichment plots of the top mouse gene set enriched in the CXCL10-positive tumor treated with NK cells and NKTR-255 compared with the CXCL10-positive tumor without the treatment

GSEA, gene set enrichment analysis; NK, natural killer; PBS, phosphate buffer solution.

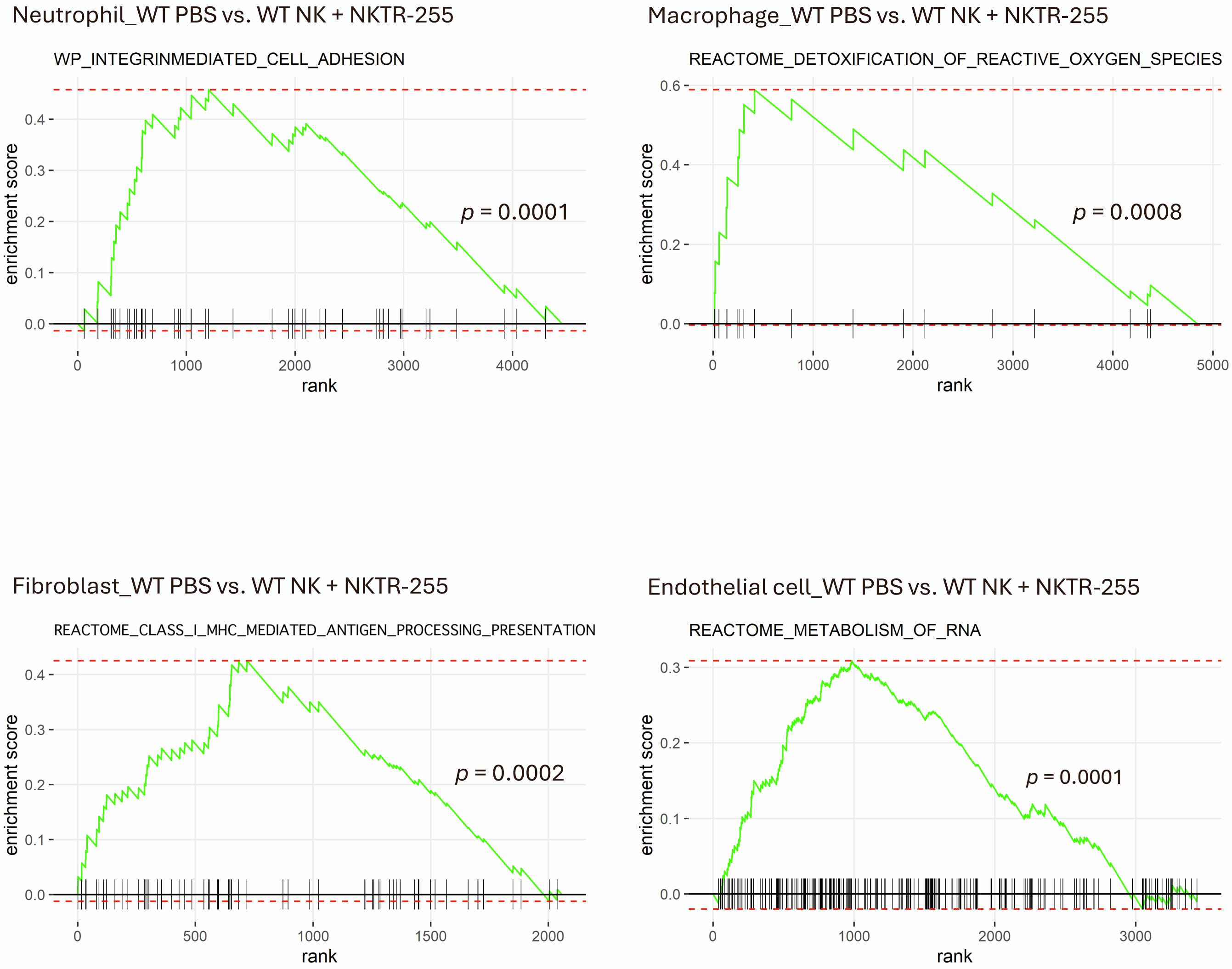

Figure S7. GSEA-enrichment plots of the top mouse gene set enriched in the WT tumor treated with NK cells and NKTR-255 compared with the WT tumor without the treatment
GSEA, gene set enrichment analysis; WT, wild-type; NK, natural killer; PBS, phosphate buffer solution.

SUPPLEMENTAL REFERENCES

1. Finck, R., Simonds, E.F., Jager, A., Krishnaswamy, S., Sachs, K., Fantl, W., Pe'er, D., Nolan, G.P., and Bendall, S.C. (2013). Normalization of mass cytometry data with bead standards. Cytometry A *83*, 483-494. 10.1002/cyto.a.22271.
2. Behbehani, G.K. (2019). Immunophenotyping by Mass Cytometry. Methods Mol Biol *2032*, 31-51. 10.1007/978-1-4939-9650-6_2.
